# Chronic Stress, Inflammation, and Colon Cancer: A CRH System-Driven Molecular Crosstalk

**DOI:** 10.3390/jcm8101669

**Published:** 2019-10-12

**Authors:** Stavroula Baritaki, Eelco de Bree, Ekaterini Chatzaki, Charalabos Pothoulakis

**Affiliations:** 1 Division of Surgery, School of Medicine, University of Crete, Heraklion, 71500 Crete, Greece; debree@med.uoc.gr; 2 Laboratory of Pharmacology, Medical School, Democritus University of Thrace, 68100 Alexandroupolis, Greece; achatzak@med.duth.gr; 3 IBD Center, Division of Digestive Diseases, David Geffen School of Medicine at UCLA, Los Angeles, CA 10833, USA; cpothoulakis@mednet.ucla.edu

**Keywords:** colorectal cancer (CRC), inflammation, stress, corticotropin releasing hormone (CRH)

## Abstract

Chronic stress is thought to be involved in the occurrence and progression of multiple diseases, via mechanisms that still remain largely unknown. Interestingly, key regulators of the stress response, such as members of the corticotropin-releasing-hormone (CRH) family of neuropeptides and receptors, are now known to be implicated in the regulation of chronic inflammation, one of the predisposing factors for oncogenesis and disease progression. However, an interrelationship between stress, inflammation, and malignancy, at least at the molecular level, still remains unclear. Here, we attempt to summarize the current knowledge that supports the inseparable link between chronic stress, inflammation, and colorectal cancer (CRC), by modulation of a cascade of molecular signaling pathways, which are under the regulation of CRH-family members expressed in the brain and periphery. The understanding of the molecular basis of the link among these processes may provide a step forward towards personalized medicine in terms of CRC diagnosis, prognosis and therapeutic targeting.

## 1. Introduction

Stress, known as a threat to maintaining organism homeostasis, is an event that most animal species experience. The idea that frequent, chronic, or excessive stress can affect the human body by increasing the risk of developing a disease is not new. According to Hippocrates, disease is nothing more than a ‘somatized’ reaction to stressful emotions. Over the years, it is becoming clear that stress is involved in the pathophysiology of a variety of diseases, including psychiatric and neurodegenerative disorders, autoimmune diseases, and inflammatory and metabolic syndromes [1,2,3].

Regulatory systems, like neural, endocrine and immune, are all involved in stress response [4,5,6]. The central stress response is mainly regulated by components of the corticotropin releasing hormone (CRH) family of neuropeptides and receptors, through their actions in the hypothalamic–pituitary–adrenal (HPA) axis [7]. In mammals, the CRH family consists of the peptides CRH, urocortin I (Ucn1), II (Ucn2), III (Ucn3), and the CRH-binding protein (CRH-bp) which, upon binding to CRH and Ucn1, mediates the peptide inactivation. The biological actions of CRH and the related peptides Ucn1, 2 and 3 are exerted by interactions with two distinct CRH receptor subtypes, CRH receptor 1 (CRHR1) and CRH receptor 2 (CRHR2). Both receptors belong to G-protein coupled receptors (GPCRs) family and signal through a cAMP-dependent mechanism. CRH and Uncs bind with distinct affinities to CRHR1 and CRHR2; Ucn1 binds both CRHR1 and CRHR2, whereas Ucn2 and 3 are selective ligands for CRHR2. The affinity of Ucn1 and 2 to CRHR2 is similar and higher than that of Ucn3 [8]. The above receptors are distributed throughout the central nervous system (CNS) and in peripheral organs, including the small and large intestines [5,8,9].

Stress mediates its downstream effects at least through disorganizing the function of the immune system in multiple directions. Experimental evidence has shown that acute stress increases resistance to infection and favors the activation of innate immunity, while chronic stress suppresses the adaptive immunity, thus making the body vulnerable to inflammatory, neoplastic, and autoimmune diseases [10]. Accordingly, excessive physical or psychological stress in humans has been reported to exert short and lasting effects on the function of many peripheral organs, including the lower gastrointestinal (GI) tract [11,12,13,14,15]. Several studies have clearly shown that stress alters intestinal functions such as gut motor and mucosal activity, visceral hypersensitivity, as well as epithelial barrier and local immune functions, via pathways involving peripheral CRH signaling [16]. These alterations have been reported to be involved in the onset and pathophysiology of chronic intestinal disorders, including inflammatory bowel disease (IBD) and irritable bowel syndrome (IBS) [9,15,17]. IBD is considered one of the predisposition factors for developing dysplasia and colorectal cancer (CRC) [18,19,20,21], mainly via modulation of the local immunological profile. A modified immune microenvironment in the colon can induce tumor initiation and progression by accelerating angiogenesis, cell proliferation and tumor cell invasiveness [18].

Here, we discuss the role of stress in the mobilization of intestinal inflammatory disorders and the development, progression and immune surveillance of neoplastic disease in the colon. Focus is given on the participation of the central and peripheral CRH system, as the core component of the molecular signaling networks that interconnect the above processes and can be targeted therapeutically.

## 2. The CRH System in the Regulation of Stress Response 

Among CRH neuropeptides, CRH is the key neuroedocrine mediator in the central HPA axis stress response [22]. In the presence of stressor stimuli of endogenous or exogenous origin, hypothalamus is stimulated to secrete CRH, which in turn promotes the release of adrenocorticotropic hormone (ACTH) from the anterior lobe of the pituitary. ACTH, then, stimulates the cortex of the adrenal glands to secrete glucocorticoids (GCs), the main class of corticosteroids. Behavioral, nervous, and/or neuroendocrine stimuli are able to induce release of hypothalamic CRH [5,8,23]. The hypothalamus and pituitary respond to HPA–axis hyperactivity (exhaustion) under chronic stress conditions, as prolonged GC production can result in a negative feedback loop and attenuation of HPA stress response, through reduction of CRH and ACTH output exposure [4,5,7,24,25].

**Figure 1:**
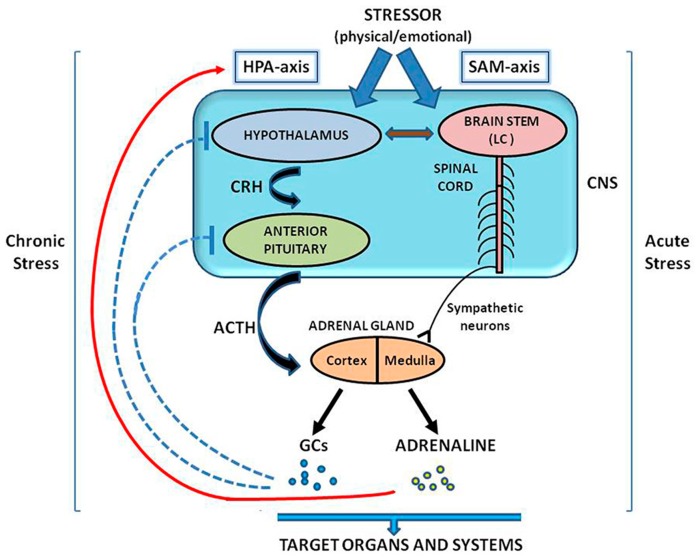
Schematic representation of the actions of HPA and SAM axes in the regulation of stress response. CRH is the key neuroendocrine mediator in HPA activation. The two axes interact with each other, through a positive bidirectional feedback loop, as described above. ACTH, adrenocorticotropic hormone; CNS, central nervous system; CRH, corticotropin releasing hormone; GCs, glucocorticoids; HPA axis, hypothalamic–pituitary–adrenal axis; LC, locus coeruleus; SAM, sympatho–adrenal–medullary axis.

Besides the central stress response of HPA-axis, stressful stimuli can also activate the medulla of adrenal glands to release catecholamines (adrenaline and noradrenaline), via stimulation of the sympathetic branch (SNS) of the autonomous nervous system (ANS), known as sympatho–adreno–medullary (SAM) axis, in an HPA-independent manner [4,5,26,27,28,29]. The action of CRH as a neurotransmitter in SAM-axis dependent stress responses is also critical [28]. Briefly, in response to stressors, CRH produced by the hypothalamus stimulates the locus coeruleus (LC), a noradrenergic center of the brainstem, to activate the α1-adrenergic receptors (α1-adrenoceptors) on preganglionic sympathetic neurons in the spinal cord [30,31]. The SNS activation triggers the medullary centers to stimulate endocrine pathways to produce catecholamines and particularly adrenaline. Adrenaline in turn acts in accelerating the sympathetic response to stressors and in promoting HPA-axis activity by stimulating CRH and ACTH production from hypothalamus and pituitary respectively, thus creating a positive bidirectional feedback loop [32]. It is generally accepted that adrenal medulla-secreted hormones mediate mainly short term (acute) stress responses, while adrenal cortex-derived hormonal signals control more prolonged (chronic) stress responses [33]. Figure 1 summarizes schematically the involvement of CNS and ANS in the regulation of stress response, as described above.

A negative feedback mechanism in the SAM axis is mediated by the central α2-adrenergic receptors, present in the LC and nucleus tractus solitaries (NTS) of the noradrenergic system of the brainstem [26,34]. CRH released in LC can also stimulate the parasympathetic preganglionic neurons in the brainstem and the spinal cord [30], leading to elevation of peripheral levels of acetylcholine, the main neurotransmitter of the other branch of ANS, the parasympathetic nervous system (PNS) [29,35]. PNS has a counter activity on SNS and usually is stimulated when the stressful situation is alleviated and no further SNS activation is needed for maintaining the physiological homeostasis [36]. 

## 3. Intestine: A Stress Target

The lower GI tract is one of the many peripheral organs targeted by stress [11,12,13,14,15]. The central neural network of the brain, which is responsible for the stress response through HPA activation, is connected to the enteric nervous system (ENS) by parasympathetic and sympathetic channels of ANS, forming the “brain-gut axis” (BGA) [37] also found in the literature as “gut–brain axis” (GBA) [38]. ANS, through the sympathetic and parasympathetic limbs, drives afferent signals coming from the lumen and transmitted through enteric, spinal and vagal pathways to central nervous system (CNS) and efferent signals from CNS to the intestinal wall [38]. The bidirectional communications among the BGA components, CNS, HPA, ANS, and ENS, involve neuro–immuno–endocrine mediators that link emotional and cognitive centers of the brain with peripheral intestinal functions, including intestinal permeability and local immunity [38]. Stress-induced activation of HPA and SAM axes may modify intestinal immune activity, via a bidirectional brain-to-immune crosstalk (neuro–immune crosstalk). The stressful signals may be translated into immune changes, while immune cell products may interfere with neuronal pathways [26,27]. CRH, either as a product of the HPA activation under stressful conditions, or, as a peripherally secreted neuropeptide, plays critical role in regulating BGA functions through neuro–immune crosstalk and consequently the intestinal immune responses [11,16,39].

### 3.1. Stress and Intestinal Inflammation. Impact of the CRH–Immune System Crosstalk

In general, stress interferes with the innate, or adaptive immunity, or even dysregulates the balance between cellular and humoral immunological responses [26,28,40]. CRH acts as a key mediator of the immunological stress response, by promoting mainly the release of corticosteroids and catecholamines upon activation of HPA and SAM axes by stressors. The immune system is vulnerable to both acute and chronic stress, mainly by being susceptible to corticosteroid and catecholamine levels. These hormones bind specific β2-adrenergic receptors present on white blood cells and have wide-ranging regulatory effects on their trafficking, proliferation and differentiation, as well as in cytokine production [28,41]. Different lymphocyte populations are characterized by variations in receptor density and ligand affinity that are considered critical for their responsiveness to stress [42,43,44]. CRH and ACTH can also alter the immune system function directly. ACTH has a high binding affinity to lymphocytes, while CRH may modulate immunity, by acting directly on secondary lymphoid organs, such as the spleen [40,45,46]. While stress, in the short run, may have at least some beneficial effects on immune cell functions and on well-being, it is chronic stress the one that puts things wrong, by increasing the risk of developing various disorders, including inflammatory and neoplastic diseases [26,28]. 

Acute and chronic stressors have been reported to increase various inflammatory markers [47,48]. A potential interaction between chronic stress and inflammatory cytokine responses to acute stress has also been reported [49,50]. Excessive stress caused by chronic bad lifestyle habits, such as poor diet or dismal life events, may inflate cortisol levels, as a result of HPA activation. High cortisol levels in turn can cause BGA disturbance and subsequent immune system dysregulation in the GI tract. The consequences include compromised food digestion and absorption by the intestine, development of indigestion and an irritated and inflamed mucosal lining. The resulting mucosal inflammation helps keeping the cortisol levels high and disorganizing the immune system, which in turn cannot respond to persistent inflammation, that becomes over the time chronic [51,52].

The gut wall is protected by a well-developed immune system and an epithelial barrier that regulates the passage of fluids, macromolecules and antigens. It also helps in limiting bacterial colonization by releasing mucus, antimicrobial peptides and immunoglobulins [16]. A physical disruption in gut epithelial barrier affects negatively the gut wall, allowing pathogens to invade the lamina propria, thus interfering with the local immunity and causing intestinal inflammation [16,53,54]. In addition, alterations in the propulsive motor function responsible for the colonic motility have been shown to trigger inflammatory conditions in the gut [11]. Experimental evidence supports a critical involvement of the central and peripheral CRH system in the regulation of both the above processes, since peripheral or systemic injection of CRH significantly promotes colonic motility and epithelial barrier permeability, thus conferring in the development of inflammatory conditions in the intestine [11,37,55]. 

The most frequent inflammatory disorder in humans that affect the lower GI tract and has been linked with higher risk for developing CRC is IBD [15,17,54,56]. Briefly, IBD is a chronic, idiopathic inflammatory condition that may exist either as ulcerative colitis (UC) or as Crohn’s disease. The incidence rate of both diseases is 10/100,000 people per year, with higher frequency in developed countries [57]. As inflammation appears to be the main component in the pathogenesis of IBD, several *in vitro* and in vivo studies have now shown that various environmental factors, including infections, together with genetic predisposition to inflammation, may converge to IBD development [58]. The prolonged inflammation results in deep ulcers and scarring of the intestinal wall, as well as in fistula formation. In general, the inflammatory process in the intestine during IBD is triggered by activation of innate and adaptive local immune responses via multiple mechanisms, including Toll-like receptor-mediated recognition of microbial structures and Th1 and Th17 activation [59]. Many of these immune-associated mechanisms can be driven by the CRH system components [59], as discussed in Section 4.1. 

Stress is also a major component of IBS, even though this intestinal inflammatory disease has not been linked to increase in CRC incidence rates [15]. IBS pathophysiology is characterized by over-sensitization of the nerves and muscles of the colon, which leads to a variety of clinical chronic symptoms. In contrast to IBD, IBS can display only low-grade inflammation in the colonic mucosa [17]. Depending on the diagnostic criteria, IBS affects globally around 11% of the general population, with women being more susceptible to the disease than men [17,58].

Although, it has long been accepted that both IBD and IBS susceptibility lies primarily in the domain of immune regulation, or epithelial integrity, only recently it became increasingly clear that neuro–immune interactions, via the participation of CRH or other neuropeptides, can substantially influence the intestinal functions and the immune responses in the GI tract. On this context, stress may have a catalytic role in mobilizing this neuro–immune interaction in gut and therefore affecting intestinal inflammation. As such, stress may influence gastrointestinal motility and ion secretion, mast cell degranulation and IFN-γ surge [60]. Nevertheless, although there is an active debate in the literature about whether stress is the dominant predisposition factor for triggering IBD onset, there is a general agreement that stress may stimulate, or enhance the inflammatory responses in the gut with, or without HPA disturbance [38,61]. These peripheral mechanisms include stress-induced increase of intestinal permeability, via vagus nerve-mediated activation of cholinergic nerves and/or atropine-sensitive transcellular and paracellular pathways [62,63,64]. The increase in epithelial barrier permeability in the bowel observed in IBD, is possible to lead to further microbial penetration and translocation, which in turn augments the inflammatory and immune response and amplifies the gut motor and sensory function dysregulation [65,66]. In addition, other stress-modulated mechanisms, such as altered secretion of chemokines and neuropeptides other than CRH (e.g. neurotensin, and substance P, vasoactive intestinal peptide), as well as activation of immune, or other origin cell subsets, may further confer to neuro–immune crosstalk, resulting in disturbance of intestinal cytokine balance and barrier integrity during the course of IBD progression [67,68] 

In the following paragraphs, we briefly discuss some of the multiple mechanisms underlying the basis of how the chronic inflammatory state, inherent in IBD, promotes malignant transformation of the colonic mucosa and progression of the disease. Special focus is given on the role of the central and peripheral CRH signaling in the above processes, as one of the critical components of the neuro–immune crosstalk that mediate the stress effects on GI tract. 

### 3.2. Inflammatory Signals and CRC Initiation and Progression: The Role of the Immune Microenvironment

It is well recognized that chronic inflammation is one of the main predisposition factors not only for cancer progression but also for contributing to neoplastic formation [18]. Patients with IBD have 2 to 6 times higher risk to develop dysplasia and colorectal cancer (CRC) compared to the general population [19,20,21], while CRC is responsible for the 10%–15% of the annual deaths among IBD patients [19]. In addition, IBD-associated CRC mainly affects young ages, with most of the cases showing a 5-year survival rate lower than 50% [19]. 

The immune imbalance in the intestinal microenvironment is considered essential for initiation and progression of cancer, including CRC [69]. A number of inflammatory cytokines present in the microenvironment of the inflamed colon is able to initiate carcinogenesis, through induction of genotoxic compounds, such as reactive oxygen species (ROS) and reactive oxygen intermediates (RNI), leading to DNA damage or activation of survival pathways, including STAT3 and NF-κB [18,56,70,71]. Furthermore, high levels of tumor infiltrated macrophage-derived MMP-9 found in CRC specimens have been associated with high risk of metastasis and poor disease outcome, as MMP-9 promotes degradation of the type IV collagen of the basement membrane [72]. 

Given the crosstalk between the gut microbiota and the immunological niche in the intestinal mucosa that determines host immunity [73], it was suggested that inflammatory disorders like IBD are tightly regulated by changes in gut microbiota composition, known as dysbiosis [74]. Indeed, microbial dysbiosis in the gut has been reported to promote BGA dysfunction, leading to local immunological imbalances and inflammation. Inflammation in turn can inflate modifications in molecular signaling networks in intestinal cells [73,75]. At the gene level, these modifications may concern epigenetic, or carcinogenic changes, which eventually pave the way to the onset and progression of gastrointestinal tract malignancies, including CRC [74,76,77]. The effects of intestinal microbial dysbiosis and inflammation on colon tumorigenesis have been studied in animal models, such as the azoxymethane/interleukin-10 knockout (AOM/Il10^-/-^) mouse model [78]. The inflammatory potential of chronic gut dysbiosis is further associated with CRC development by facilitating cell proliferation and providing a microenvironment that supports alterations in stem cell dynamics and biosynthesis of toxic and genotoxic metabolites that eventually affect the host metabolism, including glycolysis [79,80]. The produced toxins may contribute to early colon tumorigenesis via multiple pathogenic mechanisms, including among others deregulation of vital transduction networks in the colonic epithelium, such as the NF-κB, Wnt and MAPK pathways, disturbance of the Th17/IL-17 axis that regulates the differentiation of myeloid cells into myeloid-derived suppressor cells, and elevation of the Treg population [81,82,83,84]. In contrast, the anti-inflammatory role of many probiotic bacteria strains in decreasing the CRC incidence has been well demonstrated. Emerging evidence derived from in vitro and in vivo studies, where *Lactobacillus rhamnosus* was used, has revealed increased colonic epithelium cell apoptosis by induction of p53 and BAX expression, modulation of cytokine-producing human dendritic cells, reduction of the pro-inflammatory gene products β-catenin and NF-κB/p65, modification of the expression of TLR2, TLR4 and TLR9 receptors, as well as enhancement of the intestinal epithelial barrier function [85,86,87,88,89].

Accordingly, findings from recent studies that have integrated clinical- and genome-based prediction methods have revealed molecular network modules that may function synergistically towards inflammation-induced carcinogenesis in the colon [90]. Using TGF-β1-transformed colonic epithelial cells and a CRISPR-Cas9 screening strategy, Guo and colleagues managed to come up with an inflammation-induced differential genetic interaction network that was characterized by opposing synergistic crosstalk among its members [90]. The observed interactions were either CRC-promoting, or CRC-suppressing, depending on the deletions in the immune, proliferation and metabolism modules [90]. These findings underline the synergistic effects that occur among the above modules and appear to be necessary for the onset and progress of dysplasia of colorectal mucosa under inflammatory conditions. 

Overall, the spectrum of the regulators and the underlying molecular mechanisms involved in chronic inflammation-induced tumorigenesis and progression seems to be quite more complex than what we know so far and therefore it needs further elucidation.

### 3.3. Distribution of Peripheral CRH Family Members in Normal and Inflamed Intestine 

Stress, either in the form of interoceptive stimulus, such as infection and inflammation, or as an exteroceptive factor (psychological or physical stress) may affect the expression of the peripheral CRH family members in the intestine [52]. 

The distribution of CRH-related peptides and receptors in normal small and large intestine differs significantly among species and tissue types and it is not limited to enteric neurons and nerve fibers [9,16,91]. Non-neuronal expression of CRH has been found in enterocrine cells in rat and human, as well as in other epithelial cells and monocytes in humans [9,16,17]. In humans, lamina propria-derived mononuclear cells are characterized by Ucn1 overexpression. In contrast to the rat colon, which is rich in Ucn2 transcript numbers along the entire duodenal-rectal region, Ucn2 mRNA levels are low, or undetectable in the murine and human colon, respectively [52,92,93,94]. Ucn3 expression has been described mainly in the enteric plexuses of the human colon, smooth muscles, endothelium, and to a lesser extend in lamina propria mononuclear cells [9]. Non-neuronal expression of CRHR1 has been reported in lamina propria cells in rat colon and in lamina propria mononuclear cells and subepithelial mast cells in human colon biopsies [9]. The expression of CRHR2 has been reported in the rectum in rat; while in human CRHR2 mRNA and protein have been detected in lamina propria-derived mononuclear and epithelial cells [9,16]. Concomitantly, CRHR2 has been also detected in enteric plexuses, the endothelium and the vascular smooth muscles in the human colon. In contrast to CRHR1, CRHR2 levels in muscle layers have been found to be decreased with gestation in an ovine fetus model [9]. 

In normal intestine, neuronal expression of CRH has been found in a moderate number of myenteric VIPergic (vasoactive intestinal peptide) neurons and in nerve fibers located in the mucosa, submucus ganglia and the circular muscle layer in the rat colon [95,96]. Ucn1 expression persists over CRH in cholinergic and serotonergic submucous and myenteric neurons of the rat colon [9,97]. In contrast to Ucn2, Ucn3 expression has been detected in human enteric neurons and glial cells [9]. Expression of CRHR1 mRNA and protein has been detected mainly in cholinergic myenteric neurons and myenteric fibers of the rat colon, which are in close proximity with Ucn1-expressing neuronal bodies [98]. In the same model, neuronal CRHR2 expression was limited to fibers of unspecified origin [9].

The expression of CRH family members also appears to differ among various inflammation-related animal models and IBD biopsies, following a species and tissue-related manner of distribution [9]. However, the upregulation and the pro-inflammatory action of CRH seem to be a hallmark in all intestinal inflammatory disorders [99,100]. In a murine *Clostridium difficile* (CD) toxin A- induced inflammation model, increased CRH and both CRHR1 and CRHR2 expressions were detected on sub-epithelial cells and lamina propria and epithelial cells, respectively. Treatment with a CRHR1 antagonist reduced inflammation, thus suggesting a pro-inflammatory role of CRHR1 in this model [101]. Similarly, SCID mice bearing a human fetal intestine graft in which inflammation had been induced by CD toxin A, showed increased colonic mucosal CRHR2 and Ucn2 levels [102]. In rat LPS-induced and peptidoglycan-polysaccharide-induced inflammation models, increased CRH mRNA expression was detected in the enteric plexuses, colonic mucosa, and submucosa and inflammatory and mesenchymal cells, respectively [103,104]. In contrast, in rat TNBS–induced colitis models, a significant decrease in CRHR2 expression was observed on myenteric neurons and macrophages during the inflammation onset, followed by significant induction of Ucn2 expression, which possibly was mediated by increased infiltration of Ucn2-expressing immune and fibroblast cells [9,94,101]. Concomitantly, significant CRHR2 downregulation in human colonic mucosa has also been detected in ulcerative colitis (UC) [105]. Furthermore, the severity of intestinal inflammation has been reported to exert critical role in the expression of CRH related peptides and receptors. Levels of mucosal cell-derived Ucn1 were found lower in biopsies from GC-treated IBD patients and higher in cases of more severe inflammation, while in CD-derived biopsies the epithelial expressions of CRHR2 and Ucn2 were increased in regions of active inflammation as compared to non-inflamed intestinal regions [9,102,106]. 

Overall, stress-induced alterations in the expression of CRH family members may critically affect peripheral CRH signaling, contributing further to brain-gut axis dysfunction and sustaining pro-and post-inflammatory responses in human intestine, as described in the following section.

## 4. Peripheral CRH-Driven Mediators of Intestinal Inflammation 

The intestinal epithelium consists of numerous cell subsets that are implicated not only in the regulation of the intestinal epithelial barrier functions and its secretory and motor activities, but also in the activation of the local innate and adaptive defense mechanisms [16]. Most of these cells and the processes they are involved with, may be affected by peripheral CRH signaling, contributing therefore negatively, or positively in the establishment of intestinal inflammatory reactions. 

Based on the literature, the activation of peripheral CRH receptors frequently results in contradictory effects, depending on the intestinal region they are expressed and act, the type of the targeted cell population and the nature and intensity of the stimulus. Depending on the inflammation status (acute or chronic), the derived signals might act differently. CRHR1 activation appears to mainly sustain and promote inflammation, while CRHR2-driven signaling seems to drive anti-inflammatory responses, especially under low availability of its selective ligand, Ucn2 [100,101,107,108,109,110,111]. This generalized pattern of peripheral CRHR1/CRH-mediated pro-inflammatory and CRHR2-dependant anti-inflammatory responses in the intestine differentiate significantly across the GI tract, and in many cases show a species and time-sensitive distribution. Characteristically, in a DSS induced colitis murine model, CRHR1 knockdown showed decreased intestinal inflammation, while CRHR2 knockdown exerted increased inflammation, thus supporting the pro-inflammatory and anti-inflammatory roles of CRHR1 and CRHR2 respectively in the intestine [108].

Below, we present an overview of the major mediators of intestinal inflammation, whose contribution to inflammatory responses has been reported to be affected by peripheral CRH signaling. Special focus is given on peripheral CRH-driven modifications in adaptive and local innate immunity, fibroblast and endothelial cell functions, enteric neuron signaling, as well as in intestinal microbiota composition.

### 4.1. Immunity 

Although for several years the intestinal inflammatory disorders, such as IBD and IBS, were considered to be neurological conditions that result from alterations in BGA, growing evidence reveals local immunological disturbances and cytokine pattern imbalances in IBD and IBS patients, boosted by peripheral CRH signaling [17,112]. Most of the *in vitro* data, derived bydifferent species, comply with a local pro-inflammatory action of peripheral CRH, via induction and release of pro-inflammatory cytokines from GI-derived immune cells [17,100,113,114] and stimulation of neutrophil chemotaxis [115]. 

In the following paragraphs, we outline the input of peripheral CRH signaling on cell populations involved in adaptive and innate immunity in the intestine and overview the contributing role of these CRH-driven alterations onn local inflammatory responses.

#### 4.1.1. Adaptive Immunity

##### B and T cells

B and T cell populations, the specific cells of adaptive immunity, are mainly found in the intestinal mucosa [116]. Their number, activation status, and secreted cytokine and antibody patterns, differentiate significantly in intestinal inflammatory conditions, thus supporting their role in local GI inflammation. IBS patients’ colonic biopsies show increased infiltration of activating CD69 expressing T cells [17,112], while in other studies the rise in T lymphocytes, following GI infection, has been associated with augmented gut permeability [117] and changes in gut motility [118]. Likewise, B cells derived from IBS patients’ blood or LPS-exposed B cells displayed elevated expression of co-stimulatory molecules such as CD80, resulting in amplified activation [119]. The number of IgA-secreting B cells, known to protect intestinal mucosal surfaces from invasion of pathogens, is reduced in the ascending colon of IBS patients [120]. 

Considerable amount of evidence has also demonstrated T and B cell ability to expressand secrete CRH, which may act locally as a pro-inflammatory mediator [121,122]. IBD is characterized for driving Th1 and Th17 lymphocyte responses, such as production of IFN-γ, TNF-α, IL-1, IL-6, IL-8, and MIP-1α which trigger and maintain inflammation in the intestine, partly through neutrophil chemotaxis [115,123]. This immunological cytokine pattern cannot be counteracted by Treg and Th2 cell-driven anti-inflammatory responses as under physiological conditions [123,124]. CRH triggers lymphocyte proliferation by inducing IL-2R overexpression and IL-1 and IL-2 secretion [125]. Mitogenic stimulation of T cells with PHA, temporarily increased CRH expression [126], while CRH release by human B and T cells is triggered by stressful stimuli such as hyperthermia, hyperosmolarity, and hypoxia and controlled by GC levels [100,121,127]. Ucn1 synthesis and release by immune cells [92,128] may further induce IL-6 release, which is linked to ERK, p38 MAP kinase, and NF-κB activation, thus evoking a local pro-inflammatory reaction [129].

In vivo findings have further corroborated the pro-inflammatory role of CRH and CRHR1 in the intestine, through direct influence on the cytokine profile secreted by the immune cells of the adaptive immunity. In rat and murine CD toxin A-induced ileal inflammation and TNBS-induced colitis models respectively, CRH knockdown significantly ablated inflammation, with a reduction in local IL-1β levels [120,130]. As such, CRH knockdown mice treated with CRH receptor antagonists, developed less severe inflammation, which was attributed to the lack of toxin A to increase substance P (SP) levels [131]. Similarly, treated CD toxin A-induced ileitis mice models also showed less inflammation, due to CRHR1-dependent decrease in TNF-α and IL-1β expression [101]. However, controversial reports on the pro-inflammatory role of CRHR1 also exist in the literature. CRHR1 agonists such as Ucn1 were shown to exert anti-inflammatory properties in endotoxemic, or TNBS-induced colitis murine models. This result was credited to a shift of Th1-driven responses towards a Th2 response, with a systemic and local upregulation of the anti-inflammatory cytokine IL-10, as well as to the promotion of Treg activity [132,133]. 

#### 4.1.2. Innate Immunity

The effects of peripheral CRH receptor signaling on the local immune system as protector of the intestinal epithelial barrier, also involve several cell types of the innate immunity, including mast cells, macrophages and cells of the intestinal epithelium, and hold an impact on their secreted cytokine patterns.

##### Dendritic Cells

Peripheral CRH targets intestinal lamina propria dendritic cells whichexpress both CRH receptors [17,113,134]. Intestinal dendritic cells, as components of the adaptive immunity, internalize luminal antigens, through endocytosis, and present them to naïve T cells located in mesenteric lymph nodes [17]. Previous studies have reported implication of the intestinal dendritic cells in the pathogenesis of IBD, based on their CRH-producing and secreting abilities upon bacterial stimulation [135,136]. CRH enhances inflammation in LPS-treated immortal JAWS II cells, a known DC cell line derived from BMDCs of a C57BL/6 p53-knockout mouse, and murine mesenteric lymph node-derived dendritic cells, via distinct CRHR1-dependent mechanisms, that include stimulation of T-cell proliferation, elevation of IL-6 and MIP-1α secretion and reduction in the anti-inflammatory IL-4 levels [17,113,134]. In addition, treatment of human monocyte-derived dendritic cells with CRH attenuated IL-18 production which has an anti-inflammatory action [137]. The increase of dendritic cell-mediated T-cell stimulation after treatment with CRH has been corroborated by many in vitro and in vivo studies; however, a CRH-mediated shift towards a Th2 cytokine response has been further demonstrated [17,138]. Interestingly, mesenteric lymph node-derived dendritic cells obtained from an acetic acid and restraint stress-IBS rat model showed an increased T cell proliferation and enhanced secretion of anti-inflammatory IL-4 and IL-9 cytokines, after co-culture with splenic CD4^+^/CD8^+^ T cells [17,138]. Concomitantly, intestinal lamina propria dendritic cells were able to stimulate CD4^+^ T cells in post-infectious IBS mice [139], while a colorectal distension restraint stress IBS rat model showed an increase in CD103+ cell number and in IL-4 and IL-9 cytokine production in the colon [17].

##### Macrophages

Beyond mast cells, peripheral CRH signaling also targets other components of the innate immune response in the intestine, such as macrophages, thusaffecting further the tissue immune homeostasis [140]. Intestinal macrophages can clear tissue-invaded pathogens by phagocytosis, while they are also able to present antigens and secrete cytokines [112]. Intestinal macrophages may express both CRHR1 and CRHR2 and secrete CRH [106,121,141]. IBD-patient derived colonic mucosa biopsies have been shown to be enriched in CRH-immuno-reactive enterochromaffin and macrophage cells, while CRH- and Ucn1-expressing mononuclear and macrophage cells were also found in the lamina propria of UC colonic biopsies [39,92,106]. 

Mucosal macrophages have been shown to modulate colonic peristaltic activity, after stimulation by intestinal epithelial cell-secreted mediators, in a CRH-dependent manner [142,143]. Although there is some controversy in the literature about the role of CRH, as it relates to intestinal macrophages and inflammation management, most of the *in vitro* findingss on CRH receptor signaling on intestinal macrophages are supportive of pro-inflammatory and anti-inflammatory actions of CRHR1/CRH and CRHR2 signaling cascades, respectively. As such, only CRH-mediated stimulation of CRHR1 has been shown to increase antigen-specific antibody response via NF-κB activation in macrophages [141]. CRH can trigger macrophages to overproduce pro-inflammatory cytokines, such as TNF-α and IL-6, most likely via a CRHR1-dependent manner, as evidenced in BALB/c mice after treatment with LPS and a CRHR1 antagonist [114]. Furthermore, CRH and/or Ucns may also enhance chemotaxis of mononuclear cells and macrophage activation by endotoxin with subsequent release of oxidative mediators and other pro-inflammatory cytokines [100,101,102,140,144,145,146,147]. Although, in vitro treatment of murine macrophages with the CRHR1 agonists CRH and Ucn1, or with Ucn2, led to inhibition of LPS-induced TNF-α release during the onset of the inflammatory response, in a non-selective receptor manner; however, at later stages a significant increase in TNF-α transcription and release was observed [17,100,146]. These findings suggest that CRH receptor signaling on local macrophages may differentiate during the course of the inflammatory response in the intestine, and that CRH-peptide-mediated modulation of inflammatory processes may be dose-dependent. In contrast, binding of only low doses of Ucn1 and Ucn2 in CRHR2 may exert anti-inflammatory functions partly via triggering macrophages to undergo apoptosis [148]. 

However, the anti-inflammatory action of CRH, as it relates to innate immunity, has also been proposed in the literature. In support to this notion, we previously reported that CRH may protect against colitis through regulation of the toll-like receptor 4 (TLR4) expression [149]. CRH, Ucn1 and Ucn2 have been implicated in inducing the transcription of TLR4 in macrophages in a CRHR2-dependent manner [140]. TLR4 is playing a fundamental role in activating the innate immune system. We showed that mice deficient in CRH were more susceptible to DSS-induced colitis, possibly due to overproduction of IL-12 and prostaglandin E2, while having significantly decreased TLR4 levels before, but not after the colitis induction. This result corroborates the anti-inflammatory effect of peripheral CRH in innate immunity-dependent colitis and its recovery phase [149]. Consistently, decreased number of CD68+ macrophages and levels of macrophage-attracting chemokines have been found in intestinal biopsies of IBS [112], while colonic biopsies from UC showed increased CRHR1 immunoreactivity in lamina propria macrophages [122].

##### Intestinal Epithelium

The intestinal epithelium is enriched by cells capable of secreting antimicrobial peptides (AMPs) and mucus, therefore contributing to host defense against luminal antigen and pathogens [150,151]. These cells are tightly sealed by firm junctions, allowing only tiny micro molecules to penetrate the epithelium, thus preserving the intestinal barrier function [100]. Their input in innate immunity and the amplification of immune response in the intestine is further mediated by the presence of TLRs on their surface and their ability to release cytokines and chemokines [17,142]. Therefore, the intestinal epithelium is a critical player in the inflammatory responses in the gut and the related disorders.

The mucus producing cells, namely Goblet cells, are present in the ileum and colon and form a coating layer over the intestinal epithelium [150]. Goblet cells from human and rat sigmoid colon are enriched in CRH and CRHR1 expression [104,122,152,153], while CRHR2 has been detected in epithelial cells of distal/sigmoid colon biopsy samples [154]. A direct action of central and peripheral CRH system in depletion of Goblet cells and mucus release, through CRHR1 signaling, has been proposed. Studies showed that stress, or CRH administration, decreased significantly the number of Goblet cells, as well as their mucus secretory potential [153,155,156,157]. At the cell–cell tight junctions in intestinal epithelium, stress-mediated CRH release reduces expression of zona-occludens-1 and increases claudin2 levels, thus augmenting intestinal permeability [158,159]. Indeed, studies in rat mucosal colonic tissues and human colon epithelial cell lines, showed that exposure to CRH decreases transepithelial resistance, increases the epithelium permeability to macromolecules, as well as induces TLR4 expression, a finding consistent with the high TLR4 expression found in peripheral blood of IBS patients [160,161,162]. In a Wistar-Kyoto rat model, peripheral CRH induced distal colon-derived ion secretion, triggered by a non-selective CRH receptor signaling [163]. Furthermore, we recently reported in vitro and in vivo findings showing that CRHR2 expression in colonic epithelium play a vital role in resolving inflammation and promoting healing in UC mice, by increasing epithelial cell proliferation and migration, as well as by reducing apoptosis and secretion of pro-inflammatory cytokines [164]. 

AMP-producing cells are mainly located inside the crypts of the small intestine and may be mast cells, epithelial cells, or Paneth cells [165]. Although, the direct involvement of peripheral CRH signaling in altering the AMP secretion by these cellshas not been yet studied, reports from human and rodents demonstrate that stress may cause dysfunction of this innate defense mechanism and this defect could participate in the pathogenesis of IBD and IBS [16,59].

##### Mast Cells

Experimental evidence has further shown that CRH, like stress, may exert pro-inflammatory peripheral effects possibly through recruitment and activation of mast cells. Mast cells express CRH receptors and are critical for immune responses and inflammatory conditions, by releasing cytokines and other pro-inflammatory mediators known to affect the integrity of intestinal epithelial barrier [166,167]. IBS patients, under psychological stress have increased numbers of colonic and jejunum-located mast cells [168]. Mice treated with cortagine, a selective CRHR1 agonist, showed increased colonic TGF-β expression known to be a potent modulator of human intestinal effector mast cell functions [169]. CRH also increased human colonic mucosa macromolecular permeability, a potentially pro-inflammatory event, via CRH receptors R1 and R2 expressed on subepithelial mast cells [63,163,170,171]. Similarly, in a porcine *ex vivo* intestinal model, CRH induced the release of TNF-α and proteases, through a mast cell-dependent mechanism, thus resulting in augmentation of the intestinal paracellular permeability and mast cell degranulation, which are all hallmarks for IBD and IBS [172,173]. Similarly to CRH effects, acute stressors in mice lead to increased IFN-γ-mediated colonic mast cell degranulation, while chronic stress increases CRH-expressing eosinophil infiltration in the jejunum, which further contributes to epithelial barrier dysfunction, through mast cell recruitment [174]. Peripheral CRH mimics the effect of stress in increasing the small intestine permeability, through the participation of mast cells [175]. CRH signaling in the ileal and colonic mucosa may recruit and activate in addition to mast cells other immune cell populations, including neutrophils, T and NK cells [16]. The latest immune cell populations may confer to bacterial penetration into the enterocytes [157,176,177]. 

CRH-induced mast cell-derived VEGF, previously reported to be associated with skin inflammatory conditions, has not been yet investigated in intestinal inflammatory disorders [167]. However, there is experimental evidence that intestinal angiogenesis may be targeted via CRHR1 and CRHR2 signaling during inflammation. In a DSS-induced colitis mouse model, only CRHR1 knockdown decreased microvascular density, via inhibition of VEGF levels, thus suggesting that CRHR1 acts pro-angiogenic, while CRHR2 has anti-angiogenic properties during intestinal inflammation [108,178].

### 4.2. Fibroblasts and Endothelial Cells

Fibroblasts and endothelial cells isolated from inflamed tissues have the ability to produce CRH [179], which might be responsible for their activation, through a CRHR2-dependant signaling [180]. The CRH/CRHR2 signaling may also influence iNOS-mediated endothelial activation during the early phase of the inflammatory response [181]. Although cell responsiveness depends on their activation rate and differentiation state, there is clear evidence for a pro-inflammatory action of CRHR2 on these cells. High expression of CRHR2 and Ucn2 has also been described in human colonic mucosal cells from IBD patients [57,102], while CRHR2 stimulation by Ucn2 in human colonocytes resulted in NF-κB pathway activation and increased secretion of IL-8 and MCP-1 [102,109]. Supportive to the pro-inflammatory action of CRHR2 signaling are in vivo findings demonstrating that CRHR2 knockdown lessen inflammation in CD toxin A-induced ileal model, via inhibition of neutrophil infiltration and reduction in chemokine levels [109]. 

### 4.3. Enteric Neurons

Given that increased colonic motility is a hallmark in IBS and IBD and stress potentiates significant changes in the motility rates of both colon and ileum, it is more than likely that stress-induced CRH signaling may have a clear effect in the function of the enteric neurons [16]. In fact, inflammatory, mesenchymal and neuronal cells derived by a peptidoglycan-induced colitis rat model, had elevated CRH expression [109,130]. Studies in rats and mice have shown that CRH and Ucn1 can increase colonic motility, via a CRHR1-dependant manner, while CRHR2 activation results in attenuation of ileal phasic contractions [154,182]. In contrast to CRHR2, CRHR1 stimulation results in cholinergic, nitrergic and serotonergic signal transmission that increases colonic propulsive motor function [16]. 

### 4.4. Gut Microbiota

Gut microbiota are critical for gut immune homeostasis and the intestinal immune response. There is clear evidence from human and animal studies that stress can cause significant alterations in the composition of gut microbiota, the so-called microbial dysbiosis [183], leading to bowel dysmotility and increased permeability, factors known to facilitate inflammation [184,185,186,187,188]. Dysbiosis of intestinal microbiota is often present in IBD and IBS patients and is considered a contributing factor to disease development and progression [189,190,191].

Therefore, treatment modalities with probiotics or specific antibiotics have been proved efficient in IBS patients by improving the bowel function [184,185,192]. Likewise, gut microbe can be also targeted by exogenous CRH, which provokes alterations in microbe composition and increased colorectal motility [193]. Given that gut microbiota may affect GI motility and intestinal permeability by interacting with muscularis macrophages and enteric neurons, one can speculate that CRH-mediated alterations in gut microbiota may also contribute in shaping inflammatory environments [143]. 

Microbiota, as a part of the microbiota–gut–brain axis, develops and functions closely to HPA axis, thus supporting the bidirectional communication of the axis members [38,194]. It is more than likely that microbiota-derived signaling molecules together with biologically active gut peptides and locally secreted neuropeptides are critically important in facilitating the crosstalk between gut and brain [38,185,194]. Therefore, changes in microbiota environment and composition may lead to HPA activation and dysregulated activities of neurotransmitter systems and local immune functions, thus initiating and/or sustaining intestinal inflammatory conditions. Although, it is still unclear whether certain neuropeptides and their cognate receptors are expressed by gut microbiota, the involvement of CRH family members as intermediate messenger molecules in the microbiota–gut–brain axis cannot be excluded [195].

Collectively, there is extensive evidence in the literature showing that the activation of peripheral CRH signaling in cells participating in the control of the intestinal defense mechanisms and secretory and motor functions, mediates the stress-induced changes in gut physiology. Autocrine, or paracrine stimulation of peripheral CRH receptors by locally secreted CRH related peptides in the gut is considered mainly pro-inflammatory, as it results in local immune cell activation. This activity makes the peripheral CRH system in the gut, in addition to central CRH signaling, a promising therapeutic target in the management of stress-induced chronic intestinal inflammatory disorders.

## 5. Inflammation and CRC Crosstalk via the CRH System

### 5.1. Peripheral CRH System and CRC 

From what it has already been mentioned, it is clear that the distribution pattern and the intra- or extra-cellular concentration of certain members of the CRH family of peptides and receptors on colonic mucosa, as well as the signaling pathways they mobilize, play a key role in regulating the intestinal inflammatory response [17,112]. However, the contribution of the peripheral CRH system on CRC development and pathophysiology, especially in response to chronic inflammation of the colonic mucosa, has not been elucidated so far. 

On 2007, Reubi and colleagues first reported lost expression of CRHR2 in a small number of colonic tissues derived from CRC patients compared to normal counterparts [110]. A follow up study failed to validate the above findings and instead showed elevated expression of CRHR2 and Ucn3 in 30 CRC tissue samples, an increase that was revealed to be significantly associated with decreased cell adhesion and enhanced cell motility [196]. Given this controversy, we recently performed further investigation on the distribution of CRH family members in a larger cohort of CRC patient samples and explored the functional impact of any expression discrepancies on CRC pathophysiology *in vitro* and in vivo, in context of intestinal inflammation. In parallel, we investigated any possible implications of CRH-driven signaling in the regulation of tumor responsiveness to endogenous anti-tumor immuno-mediated cytotoxic responses. 

Our novel findings, which are discussed below, are the first to provide clear evidence for the role of the CRH system, as a direct linker between chronic inflammation in the colonic mucosa and CRC growth, spread and immunoescape. 

#### 5.1.1. Negative Contribution of the Peripheral CRHR2/Ucn2 Signaling in CRC Progression and Metastatic Potential

**Figure 2:**
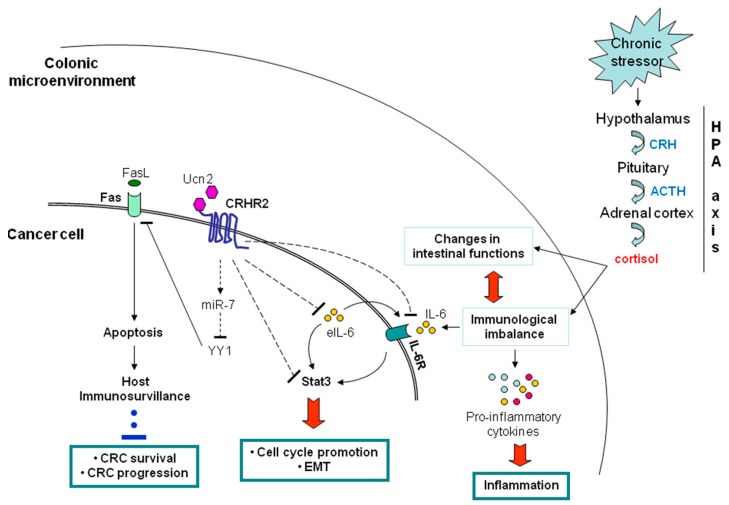
A proposed mechanism for the anti-tumoridical activity of peripheral CRHR2/Ucn2 signaling in inflammation-related colorectal cancer (CRC). CRHR2 expression has been found significantly diminished in CRC cells. Colonic inflammation, which is often induced by chronic stressors, is characterized by accumulation of several pro-inflammatory cytokines, including IL-6, in the local microenvironment, produced either by infiltrated immune cells, or cancer cells, when present. Experimental evidence demonstrates that when CRHR2 is expressed on cancer cell surface and activated by Ucn2, it can sufficiently inhibit the endogenous expression of IL-6 and its receptor, resulting in reduction of IL-6 mediated Stat3-phosphorylation and activation. Stat3 inhibition in CRC cells, affects negatively the expression of STAT-3 target genes involved in cell cycle promotion and EMT, thus repressing CRC growth and metastasis. In addition, CRHR2/Ucn2 signaling was shown to reverse tumor resistance to Fas-mediated apoptosis in CRC cells by inducing miR-7, a suppressor of YY1, which normally acts as a transcriptional repressor of Fas in cancer cells. The proposed mechanism is an example of how the peripheral CRH system can effectively mediate and control the crosstalk among molecular networks involved in stress-induced inflammation and CRC pathophysiology. Dotted lines represent the CRHR2/Ucn2-mediated effects on signal transduction pathways in CRC cells. eIL-6, endogenous tumor produced IL-6; EMT, epithelial to mesencymal transition; CRHR2, corticotrophin releasing hormone receptor 2.

Expression studies in a large number of normal and CRC human tissues and cell lines revealed that among the CRH family members, CRC samples had significantly reduced or lost CRHR2 expression and elevated levels of its specific agonist, Ucn2 [197]. These findings corroborate the CRHR2 downregulation and Ucn2 elevation observed in vivo in TNBS-induced colitis animal models and *ex vivo* in UC human biopsies, in the late stages of inflammation [9,94,101]. It cannot be excluded that the observed CRHR2 elimination might be a response compensating for UCN2 upregulation, through an unknown regulatory mechanism [9]. In our experimental model, ectopic induction of CRHR2 in CRC cell lines resulted in dramatic inhibition of the endogenous Ucn2 levels, while the receptor activation by exogenous Ucn2 reduced critically the tumor survival and expansion in vitro and in vivo in xenograft mouse models. The underlying molecular mechanisms involved disruption of the inflammatory signals and Stat3 activation in the CRC cell lines, as well as suppression of the oncogenic epithelial to mesenchymal transition (EMT), known to be necessary for the initiation of the metastatic process [197] (Figure 2). An overview of the proposed mechanism of CRHR2/Ucn2 involvement in inflammation-related CRC is depicted in Figure 2. The prognostic significance of the CRHR2 levels in CRC was evident by statistically significant positive associations established between CRHR2 expression and overall patient survival, attributed in part to a lower risk for distant metastasis. 

CRC initiation and progression are closely affected by the extent and duration of the mucosal inflammation, which is supported by inflammatory immune cell infiltration and by the tumor cells themselves. These cells have the potential to release a variety of tumor-promoting cytokines, such as IL-6 and mitogens [198]. We showed that decreased expression or loss of CRHR2 in CRC cell lines coincides with increased expression of IL-6 and its receptor IL-6R, while CRHR2 and IL-6R expression levels in CRC tissues were inversely correlated. Given that Ucn2 has been implicated in supporting topical inflammation, through induction of IL-6 [199], we hypothesized that under minimal CRHR2 expression the high Ucn2 levels detected in CRC tissues and cell lines, might contribute in maintaining and/or promoting IL-6 expression, thus serving as an inflammatory stimulus supportive for IBD-related carcinogenesis. Accordingly, endogenous Ucn2 levels were significantly reduced after ectopic induction of CRHR2 in CRC cells, whereas Ucn2 silencing in wild type CRC cell lines resulted in increased CRHR2 levels and reduced IL-6 secretion [197]. These findings support our hypothesis and suggest that CRHR2^high^ CRC tumors with low Ucn2 expression levels may be more resistant to inflammatory signals such as IL-6 provided and acting via autocrine, or paracrine pathways. 

This last notion was further supported by findings demonstrating significant elimination of Stat3 activation in CRHR2-overexpressing CRC cells after external administration of IL-6. The aberrant and persistent Stat3 activation, by cytokines like IL-6 and IL-10, is known to function as a molecular link between intestinal inflammation and colorectal tumorigenesis [200]. Stat3 mediates transcriptional responses favoring CRC survival, proliferation, angiogenesis, and therefore an overall poor patient survival [201,202]. We first identified Stat3 as a novel downstream target of CRHR2/Ucn2 signaling in CRC, by demonstrating significant suppression of IL-6-mediated Stat3 (Tyr703) phosphorylation after CRHR2 induction and activation by Ucn2, followed by decreased cell proliferation *in vitro* and diminished tumor growth in vivo. CRHR2/Ucn2 signaling was further shown to target negatively key IL-6-dependent oncogenic EMT-inducers and angiogenic factors, such as VEGF, *in vitro* and in vivo, possibly through IL-6/Stat-3 signaling inhibition. Therefore the above findings suggest a decisive role of CRHR2/Ucn2 signaling in the regulation of the metastatic potential and expansion of the CRC cells. 

Contrarily, an oncogenic role has been described for the CRHR1/CRH signaling in a colitis associated-cancer mouse model. Opposed to CRHR2/Ucn2 signaling, CRHR1 activation by CRH promoted selectively colon cancer cell proliferation through an IL-6/JAK2/STAT3-depended mechanism and induced angiogenesis via VEGF up regulation [203,204]. At the same line, other studies have highlighted the type of ligand that activate CRHR2 as a critical determinant of the impact that CRHR2 signaling may have on the stress-induced epithelial alterations that eventually can mediate mucosal barrier dysfunction, worsening of mucosal inflammation and malignant cell transformation [205,206].

Overall, our findings discussed above provide clear evidence that the distribution of CRHR2 in the inflamed colonic mucosa and the local availability of its selective agonist Ucn2, via an autocrine, or paracrine axis, determine in a large part the CRC response to inflammatory signals that support its survival, growth, and metastatic behavior. Therefore, CRHR2 and its agonist Ucn2 may be claimed as putative prognostic factors and therapeutic targets in CRC. 

#### 5.1.2. Contribution of CRHR2/Ucn2 Signaling in CRC Immune Surveillance

It is known that CRC often develops not only resistance to conventional chemotherapy but also to immuno-mediated cytotoxic signals, leading to tumor immuno-escape. The role of CRH family members in cancer immunobiology is poorly elucidated. Our ex vivo studies have showed that CRC compared to control tissues have reduced Fas expression, which is positively correlated with lost CRHR2 transcript levels, poor tumor differentiation and high risk for distant metastasis [207]. To examine the contribution of the CRHR2 downregulation in CRC immuno-resistance, we compared the apoptotic responses of CRHR2-overexpressing CRC cell lines and those of wild type cells after stimulation with Ucn2 and CH11 antibody, a FasL analogCH11. Induction of CRHR2/Ucn2 signaling revealed a significantly elevated sensitivity of CRC cells to CH11-mediated apoptosis, via elevation of the surface Fas expression. Further analysis of the underlying molecular mechanism demonstrated CRHR2/Ucn2-dependent inhibition of the Fas transcriptional repressor YY1, via induction of miR-7, an endogenous suppressor of YY1 [208] (Figure 2). Exogenous modulation of miR-7 levels and CRHR2 activation in various CRC cell lines had opposing effects on YY1 and Fas expressions and cell sensitivity to CH11-mediated apoptosis, thus suggesting specificity of CRHR2/Ucn2 inhibitory action on YY1, via miR-7 [207]. The inverse correlation between YY1 and Fas expression levels was further validated in human CRC tissue arrays. In addition, YY1 mRNA overexpression in patient samples was positively correlated with both advanced CRC grade and high risk for distant metastasis [207].

## 6. Conclusions––What Is Next?

Although for many years body stress, chronic inflammation, and carcinogenicity in the intestine have been studied as individual events, recent findings suggest that they are the vertices of a triangle with an inextricable connection among them. There are now strong indications that the pattern of expression of key stress regulators, such as members of the CRH family, in peripheral organs like the colon, plays a critical role not only in signal transmission via the HPA axis, but also in the type of the response that the peripheral organ exerts under inflammatory stimuli, as well as in the organ’s predisposition for cancerous excretion and rapid disease progression. Therefore, the CRH system functions as an orchestrator of a peripheral response to body stress that can be ’somatized’ as a chronic inflammation, and/or act as an ideal background for carcinogenesis. For example, the complete lack or low expression of the CRHR2 receptor in the large intestine works positively in establishing, maintaining and enhancing an inflammatory microenvironment in the organ, while promoting carcinogenesis and a subsequent aggressive nature of the disease. Characteristically, based on our reported findings, CRC patients with poor CRHR2 expression levels, had the worst prognosis for distant metastases and 5-year overall survival [197].

A reasonable question which is raised here is whether and how we can use the current knowledge in the era of identifying critical regulators of the interaction among emotional stress, chronic inflammation and carcinogenesis. Undoubtedly, we have only touched the foot of the mountain and still need a lot of uphill road to clarify all the involved mechanisms and the underlying signaling paths that feed this interaction. However, identifying the signaling pathway CRHR2/Ucn2 as a critical negative regulator of colonic inflammation and malignancy, allows us to begin thinking about the inclusion of low CRHR2 expression levels in the colon in the list of novel putative prognostic indicators for sustaining chronic inflammation and promoting cancer development and aggressiveness in the organ. At the same time, selective induction of CRHR2/Ucn2 signaling activityin the large intestine of patients with chronic inflammation, or in those predisposed to develop, by using targeted drugs, may hold a therapeutic impact in the resolution of the inflammation and eliminate the chances the benign disease to progress to malignancy.

## References

[1] Zänkert S., Bellingrath S., Wüst S., Kudielka B.M. (2019). HPA axis responses to psychological challenge linking stress and disease: What do we know on sources of intra- and interindividual variability?. Psychoneuroendocrinology.

[2] Afrisham R., Paknejad M., Soliemanifar O., Sadegh-Nejadi S., Meshkani R., Ashtary-Larky D. (2019). The Influence of Psychological Stress on the Initiation and Progression of Diabetes and Cancer. Int. J. Endocrinol. Metab..

[3] Sharif K., Watad A., Coplan L., Lichtbroun B., Krosser A., Lichtbroun M., Bragazzi N.L., Amital H., Afek A., Shoenfeld Y. (2018). The role of stress in the mosaic of autoimmunity: An overlooked association. Autoimmun. Rev..

[4] Myers B., McKlveen J.M., Herman J.P. (2012). Neural Regulation of the Stress Response: The Many Faces of Feedback. Cell. Mol. Neurobiol..

[5] Smith S.M., Vale W.W. (2006). The role of the hypothalamic–pituitary–adrenal axis in neuroendocrine responses to stress. Dialogues Clin. Neurosci..

[6] Godoy L.D., Rossignoli M.T., Delfino-Pereira P., Garcia-Cairasco N., de Lima Umeoka E.H. (2018). A Comprehensive Overview on Stress Neurobiology: Basic Concepts and Clinical Implications. Front. Behav. Neurosci..

[7] Herman J.P., McKlveen J.M., Ghosal S., Kopp B., Wulsin A., Makinson R., Scheimann J., Myers B. (2016). Regulation of the Hypothalamic-Pituitary-Adrenocortical Stress Response. Compr. Physiol..

[8] Bale TL V.W. (2004). CRF and CRF receptors: Role in stress responsivity and other behaviors. Annu. Rev. Pharmacol. Toxicol..

[9] Buckinx R., Adriaensen D., Van Nassauw L., Timmermans J.-P. (2011). Corticotrophin-releasing factor, related peptides, and receptors in the normal and inflamed gastrointestinal tract. Front. Neurosci..

[10] FS D. (2008). Enhancing versus Suppressive Effects of Stress on Immune Function: Implications for Immunoprotection versus Immunopathology. Allergy Asthma Clin. Immunol..

[11] Stengel A., Taché Y. (2009). Neuroendocrine control of the gut during stress: Corticotropin-releasing factor signaling pathways in the spotlight. Annu. Rev. Physiol..

[12] Taché Y., Kiank C., Stengel A. (2009). A role for corticotropin-releasing factor in functional gastrointestinal disorders. Curr. Gastroenterol. Rep..

[13] Binder E.B., Nemeroff C.B. (2010). The CRF system, stress, depression and anxiety-insights from human genetic studies. Mol. Psychiatry.

[14] Taché Y., Bonaz B. (2007). Corticotropin-releasing factor receptors and stress-related alterations of gut motor function. J. Clin. Investig..

[15] Collins S.M. (2001). Stress and the Gastrointestinal Tract IV. Modulation of intestinal inflammation by stress: Basic mechanisms and clinical relevance. Am. J. Physiol. Gastrointest. Liver Physiol..

[16] Larauche M., Kiank C., Tache Y. (2009). Corticotropin releasing factor signaling in colon and ileum: Regulation by stress and pathophysiological implications. J. Physiol. Pharmacol..

[17] Chatoo M., Li Y., Ma Z., Coote J., Du J., Chen X. (2018). Involvement of Corticotropin-Releasing Factor and Receptors in Immune Cells in Irritable Bowel Syndrome. Front. Endocrinol..

[18] Grivennikov S.I., Greten F.R., Karin M. (2010). Immunity, inflammation, and cancer. Cell.

[19] Keller D.S., Windsor A., Cohen R., Chand M. (2019). Colorectal cancer in inflammatory bowel disease: Review of the evidence. Tech. Coloproctol..

[20] Zisman T.L., Rubin D.T. (2008). Colorectal cancer and dysplasia in inflammatory bowel disease. World J. Gastroenterol..

[21] Mattar M.C., Lough D., Pishvaian M.J., Charabaty A. (2011). Current management of inflammatory bowel disease and colorectal cancer. Gastrointest. Cancer Res..

[22] Vale W., Spiess J., Rivier C., Rivier J. (1981). Characterization of a 41-residue ovine hypothalamic peptide that stimulates secretion of corticotropin and beta-endorphin. Science.

[23] Aguilera G. (2011). Regulation of the hypothalamic–pituitary–adrenal axis by neuropeptides. Horm. Mol. Biol. Clin. Investig..

[24] Keller-Wood M.E., Dallman M.F. (1984). Corticosteroid inhibition of ACTH secretion. Endocr. Rev..

[25] Gaffey A.E., Bergeman C.S., Clark L.A., Wirth M.M. (2016). Aging and the HPA axis: Stress and resilience in older adults. Neurosci. Biobehav. Rev..

[26] Segerstrom S.C., Miller G.E. (2004). Psychological stress and the human immune system: A meta-analytic study of 30 years of inquiry. Psychol. Bull..

[27] Chrousos G.P. (2009). Stress and disorders of the stress system. Nat. Rev. Endocrinol..

[28] Dragoş D., Tănăsescu M.D. (2010). The effect of stress on the defense systems. J. Med. Life.

[29] Chrousos G.P., Gold P.W. (1992). The concepts of stress and stress system disorders. Overview of physical and behavioral homeostasis. JAMA.

[30] Jones B.E., Yang T.Z. (1985). The efferent projections from the reticular formation and the locus coeruleus studied by anterograde and retrograde axonal transport in the rat. J. Comp. Neurol..

[31] Lewis D.I., Coote J.H. (1990). Excitation and inhibition of rat sympathetic preganglionic neurones by catecholamines. Brain Res..

[32] Reiche E.M.V., Nunes S.O.V., Morimoto H.K. (2004). Stress, depression, the immune system, and cancer. Lancet Oncol..

[33] Tsigos C., Kyrou I., Kassi E., Chrousos G.P., Feingold K.R., Anawalt B., Boyce A., Chrousos G., Dungan K., Grossman A., Hershman J.M., Kaltsas G., Koch C., Kopp P. (2000). Stress, Endocrine Physiology and Pathophysiology.

[34] Isaac L. (1980). Clonidine in the central nervous system: Site and mechanism of hypotensive action. J. Cardiovasc. Pharmacol..

[35] Wank S.A. (1995). Cholecystokinin receptors. Am. J. Physiol..

[36] McCorry L.K. (2007). Physiology of the autonomic nervous system. Am. J. Pharm. Educ..

[37] Ducarouge B., Muriel Jacquier S. (2011). Stress neuromediators are key regulators of the intestinal barrier: Link to inflammation and cancer. Trends Cell Mol. Biol..

[38] Carabotti M., Scirocco A., Maselli M.A., Severi C. (2015). The gut–brain axis: Interactions between enteric microbiota, central and enteric nervous systems. Ann. Gastroenterol..

[39] Gross K.J., Pothoulakis C. (2007). Role of neuropeptides in inflammatory bowel disease. Inflamm. Bowel Dis..

[40] Ader R., Cohen N., Felten D. (1995). Psychoneuroimmunology: Interactions between the nervous system and the immune system. Lancet.

[41] Blalock J.E. (1994). The syntax of immune-neuroendocrine communication. Immunol. Today.

[42] Anstead M.I., Hunt T.A., Carlson S.L., Burki N.K. (1998). Variability of peripheral blood lymphocyte beta-2-adrenergic receptor density in humans. Am. J. Respir. Crit. Care Med..

[43] Landmann R. (1992). Beta-adrenergic receptors in human leukocyte subpopulations. Eur. J. Clin. Investig..

[44] Maisel A.S., Fowler P., Rearden A., Motulsky H.J., Michel M.C. (1989). A new method for isolation of human lymphocyte subsets reveals differential regulation of beta-adrenergic receptors by terbutaline treatment. Clin. Pharmacol. Ther..

[45] Tsagarakis S., Grossman A. (1994). Corticotropin-releasing hormone: Interactions with the immune system. Neuroimmunomodulation.

[46] Fridmanis D., Roga A., Klovins J. (2017). ACTH Receptor (MC2R) Specificity: What Do We Know About Underlying Molecular Mechanisms?. Front. Endocrinol..

[47] Weik U., Herforth A., Kolb-Bachofen V., Deinzer R. (2008). Acute stress induces proinflammatory signaling at chronic inflammation sites. Psychosom. Med..

[48] Maes M., Song C., Lin A., De Jongh R., Van Gastel A., Kenis G., Bosmans E., De Meester I., Benoy I., Neels H. (1998). The effects of psychological stress on humans: Increased production of pro-inflammatory cytokines and a Th1-like response in stress-induced anxiety. Cytokine.

[49] Brydon L., Edwards S., Mohamed-Ali V., Steptoe A. (2004). Socioeconomic status and stress-induced increases in interleukin-6. Brain Behav. Immun..

[50] Won E., Kim Y.-K. (2016). Stress, the Autonomic Nervous System, and the Immune-kynurenine Pathway in the Etiology of Depression. Curr. Neuropharmacol..

[51] Stephens M.A.C., Wand G. (2012). Stress and the HPA axis: Role of glucocorticoids in alcohol dependence. Alcohol Res..

[52] Bhatia V., Tandon R.K. (2005). Stress and the gastrointestinal tract. J. Gastroenterol. Hepatol..

[53] Turner J.R. (2009). Intestinal mucosal barrier function in health and disease. Nat. Rev. Immunol..

[54] Groschwitz K.R., Hogan S.P. (2009). Intestinal barrier function: Molecular regulation and disease pathogenesis. J. Allergy Clin. Immunol..

[55] Fukudo S., Nomura T., Hongo M. (1998). Impact of corticotropin-releasing hormone on gastrointestinal motility and adrenocorticotropic hormone in normal controls and patients with irritable bowel syndrome. Gut.

[56] Grivennikov S.I. (2013). Inflammation and colorectal cancer: Colitis-associated neoplasia. Semin. Immunopathol..

[57] Paschos K.A., Kolios G., Chatzaki E. (2009). The corticotropin-releasing factor system in inflammatory bowel disease: Prospects for new therapeutic approaches. Drug Discov. Today.

[58] Mearin F., Lacy B.E., Chang L., Chey W.D., Lembo A.J., Simren M., Spiller R. (2016). Bowel Disorders. Gastroenterology.

[59] Matricon J., Barnich N., Ardid D. (2010). Immunopathogenesis of inflammatory bowel disease. Self. Nonself..

[60] Demaude J., Salvador-Cartier C., Fioramonti J., Ferrier L., Bueno L. (2006). Phenotypic changes in colonocytes following acute stress or activation of mast cells in mice: Implications for delayed epithelial barrier dysfunction. Gut.

[61] Radek K.A. (2010). Antimicrobial anxiety: The impact of stress on antimicrobial immunity. J. Leukoc. Biol..

[62] Costantini T.W., Bansal V., Peterson C.Y., Loomis W.H., Putnam J.G., Rankin F., Wolf P., Eliceiri B.P., Baird A., Coimbra R. (2010). Efferent vagal nerve stimulation attenuates gut barrier injury after burn: Modulation of intestinal occludin expression. J. Trauma.

[63] Santos J., Saunders P.R., Hanssen N.P., Yang P.C., Yates D., Groot J.A., Perdue M.H. (1999). Corticotropin-releasing hormone mimics stress-induced colonic epithelial pathophysiology in the rat. Am. J. Physiol..

[64] Saunders P.R., Hanssen N.P., Perdue M.H. (1997). Cholinergic nerves mediate stress-induced intestinal transport abnormalities in Wistar-Kyoto rats. Am. J. Physiol..

[65] Yu L.C.-H., Wang J.-T., Wei S.-C., Ni Y.-H. (2012). Host-microbial interactions and regulation of intestinal epithelial barrier function: From physiology to pathology. World J. Gastrointest. Pathophysiol..

[66] Pastorelli L., De Salvo C., Mercado J.R., Vecchi M., Pizarro T.T. (2013). Central role of the gut epithelial barrier in the pathogenesis of chronic intestinal inflammation: Lessons learned from animal models and human genetics. Front. Immunol..

[67] Margolis K.G., Gershon M.D. (2009). Neuropeptides and inflammatory bowel disease. Curr. Opin. Gastroenterol..

[68] Mashaghi A., Marmalidou A., Tehrani M., Grace P.M., Pothoulakis C., Dana R. (2016). Neuropeptide substance P and the immune response. Cell. Mol. Life Sci..

[69] Balkwill F., Mantovani A. (2001). Inflammation and cancer: Back to Virchow?. Lancet.

[70] Meira L.B., Bugni J.M., Green S.L., Lee C.-W., Pang B., Borenshtein D., Rickman B.H., Rogers A.B., Moroski-Erkul C.A., McFaline J.L. (2008). DNA damage induced by chronic inflammation contributes to colon carcinogenesis in mice. J. Clin. Investig..

[71] Markman J.L., Shiao S.L. (2015). Impact of the immune system and immunotherapy in colorectal cancer. J. Gastrointest. Oncol..

[72] Zeng Z.S., Huang Y., Cohen A.M., Guillem J.G. (1996). Prediction of colorectal cancer relapse and survival via tissue RNA levels of matrix metalloproteinase-9. J. Clin. Oncol..

[73] Cianci R., Franza L., Schinzari G., Rossi E., Ianiro G., Tortora G., Gasbarrini A., Gambassi G., Cammarota G. (2019). The Interplay between Immunity and Microbiota at Intestinal Immunological Niche: The Case of Cancer. Int. J. Mol. Sci..

[74] Zou S., Fang L., Lee M.-H. (2018). Dysbiosis of gut microbiota in promoting the development of colorectal cancer. Gastroenterol. Rep..

[75] Mandal P. (2018). Molecular mechanistic pathway of colorectal carcinogenesis associated with intestinal microbiota. Anaerobe.

[76] Chen C.-C., Lin W.-C., Kong M.-S., Shi H.N., Walker W.A., Lin C.-Y., Huang C.-T., Lin Y.-C., Jung S.-M., Lin T.-Y. (2012). Oral inoculation of probiotics *Lactobacillus acidophilus* NCFM suppresses tumour growth both in segmental orthotopic colon cancer and extra-intestinal tissue. Br. J. Nutr..

[77] Gagnière J., Raisch J., Veziant J., Barnich N., Bonnet R., Buc E., Bringer M.-A., Pezet D., Bonnet M. (2016). Gut microbiota imbalance and colorectal cancer. World J. Gastroenterol..

[78] Rothemich A., Arthur J.C. (2019). The Azoxymethane/Il10^−/−^ Model of Colitis-Associated Cancer (CAC). Methods Mol. Biol..

[79] Cani P.D., Plovier H., Van Hul M., Geurts L., Delzenne N.M., Druart C., Everard A. (2016). Endocannabinoids--at the crossroads between the gut microbiota and host metabolism. Nat. Rev. Endocrinol..

[80] Tsilimigras M.C.B., Fodor A., Jobin C. (2017). Carcinogenesis and therapeutics: The microbiota perspective. Nat. Microbiol..

[81] Boleij A., Hechenbleikner E.M., Goodwin A.C., Badani R., Stein E.M., Lazarev M.G., Ellis B., Carroll K.C., Albesiano E., Wick E.C. (2015). The Bacteroides fragilis toxin gene is prevalent in the colon mucosa of colorectal cancer patients. Clin. Infect. Dis..

[82] Geis A.L., Fan H., Wu X., Wu S., Huso D.L., Wolfe J.L., Sears C.L., Pardoll D.M., Housseau F. (2015). Regulatory T-cell Response to Enterotoxigenic Bacteroides fragilis Colonization Triggers IL17-Dependent Colon Carcinogenesis. Cancer Discov..

[83] Grivennikov S., Karin E., Terzic J., Mucida D., Yu G.-Y., Vallabhapurapu S., Scheller J., Rose-John S., Cheroutre H., Eckmann L. (2009). IL-6 and Stat3 are required for survival of intestinal epithelial cells and development of colitis-associated cancer. Cancer Cell.

[84] Thiele Orberg E., Fan H., Tam A.J., Dejea C.M., Destefano Shields C.E., Wu S., Chung L., Finard B.B., Wu X., Fathi P. (2017). The myeloid immune signature of enterotoxigenic Bacteroides fragilis-induced murine colon tumorigenesis. Mucosal Immunol..

[85] Harusato A., Viennois E., Etienne-Mesmin L., Matsuyama S., Abo H., Osuka S., Lukacs N.W., Naito Y., Itoh Y., Li J.-D. (2019). Early-Life Microbiota Exposure Restricts Myeloid-Derived Suppressor Cell-Driven Colonic Tumorigenesis. Cancer Immunol. Res..

[86] Evrard B., Coudeyras S., Dosgilbert A., Charbonnel N., Alamé J., Tridon A., Forestier C. (2011). Dose-dependent immunomodulation of human dendritic cells by the probiotic Lactobacillus rhamnosus Lcr35. PLoS ONE.

[87] Kuugbee E.D., Shang X., Gamallat Y., Bamba D., Awadasseid A., Suliman M.A., Zang S., Ma Y., Chiwala G., Xin Y. (2016). Structural Change in Microbiota by a Probiotic Cocktail Enhances the Gut Barrier and Reduces Cancer via TLR2 Signaling in a Rat Model of Colon Cancer. Dig. Dis. Sci..

[88] Dong L., Li J., Liu Y., Yue W., Luo X. (2012). Toll-like receptor 2 monoclonal antibody or/and Toll-like receptor 4 monoclonal antibody increase counts of *Lactobacilli* and *Bifidobacteria* in dextran sulfate sodium-induced colitis in mice. J. Gastroenterol. Hepatol..

[89] Gamallat Y., Meyiah A., Kuugbee E.D., Hago A.M., Chiwala G., Awadasseid A., Bamba D., Zhang X., Shang X., Luo F. (2016). Lactobacillus rhamnosus induced epithelial cell apoptosis, ameliorates inflammation and prevents colon cancer development in an animal model. Biomed. Pharmacother..

[90] Guo Y., Bao C., Ma D., Cao Y., Li Y., Xie Z., Li S. (2019). Network-Based Combinatorial CRISPR-Cas9 Screens Identify Synergistic Modules in Human Cells. ACS Synth. Biol..

[91] Taché Y., Perdue M.H. (2004). Role of peripheral CRF signalling pathways in stress-related alterations of gut motility and mucosal function. Neurogastroenterol. Motil..

[92] Muramatsu Y., Fukushima K., Iino K., Totsune K., Takahashi K., Suzuki T., Hirasawa G., Takeyama J., Ito M., Nose M. (2000). Urocortin and corticotropin-releasing factor receptor expression in the human colonic mucosa. Peptides.

[93] Chen A., Blount A., Vaughan J., Brar B., Vale W. (2004). Urocortin II gene is highly expressed in mouse skin and skeletal muscle tissues: Localization, basal expression in corticotropin-releasing factor receptor (CRFR) 1- and CRFR2-null mice, and regulation by glucocorticoids. Endocrinology.

[94] Chang J., Hoy J.J., Idumalla P.S., Clifton M.S., Pecoraro N.C., Bhargava A. (2007). Urocortin 2 expression in the rat gastrointestinal tract under basal conditions and in chemical colitis. Peptides.

[95] Sand E., Themner-Persson A., Ekblad E. (2011). Corticotropin releasing factor-distribution in rat intestine and role in neuroprotection. Regul. Pept..

[96] Liu S., Gao N., Hu H.-Z., Wang X., Wang G.-D., Fang X., Gao X., Xia Y., Wood J.D. (2006). Distribution and chemical coding of corticotropin-releasing factor-immunoreactive neurons in the guinea pig enteric nervous system. J. Comp. Neurol..

[97] Kimura T., Amano T., Uehara H., Ariga H., Ishida T., Torii A., Tajiri H., Matsueda K., Yamato S. (2007). Urocortin I is present in the enteric nervous system and exerts an excitatory effect via cholinergic and serotonergic pathways in the rat colon. Am. J. Physiol. Gastrointest. Liver Physiol..

[98] Liu S., Ren W., Qu M.-H., Bishop G.A., Wang G.-D., Wang X.-Y., Xia Y., Wood J.D. (2010). Differential actions of urocortins on neurons of the myenteric division of the enteric nervous system in guinea pig distal colon. Br. J. Pharmacol..

[99] Li B., Lee C., Filler T., Hock A., Wu R.Y., Li Q., Chen S., Koike Y., Ip W., Chi L. (2017). Inhibition of corticotropin-releasing hormone receptor 1 and activation of receptor 2 protect against colonic injury and promote epithelium repair. Sci. Rep..

[100] Kiank C., Taché Y., Larauche M. (2010). Stress-related modulation of inflammation in experimental models of bowel disease and post-infectious irritable bowel syndrome: Role of corticotropin-releasing factor receptors. Brain Behav. Immun..

[101] Wlk M., Wang C.C., Venihaki M., Liu J., Zhao D., Anton P.M., Mykoniatis A., Pan A., Zacks J., Karalis K. (2002). Corticotropin-releasing hormone antagonists possess anti-inflammatory effects in the mouse ileum. Gastroenterology.

[102] Moss A.C., Anton P., Savidge T., Newman P., Cheifetz A.S., Gay J., Paraschos S., Winter M.W., Moyer M.P., Karalis K. (2007). Urocortin II mediates pro-inflammatory effects in human colonocytes via corticotropin-releasing hormone receptor 2alpha. Gut.

[103] van Tol E.A., Petrusz P., Lund P.K., Yamauchi M., Sartor R.B. (1996). Local production of corticotropin releasing hormone is increased in experimental intestinal inflammation in rats. Gut.

[104] Yuan P.-Q., Wu S.V., Wang L., Taché Y. (2010). Corticotropin releasing factor in the rat colon: Expression, localization and upregulation by endotoxin. Peptides.

[105] Chatzaki E., Anton P.A., Million M., Lambropoulou M., Constantinidis T., Kolios G., Taché Y., Grigoriadis D.E. (2013). Corticotropin-releasing factor receptor subtype 2 in human colonic mucosa: Down-regulation in ulcerative colitis. World J. Gastroenterol..

[106] Saruta M., Takahashi K., Suzuki T., Torii A., Kawakami M., Sasano H. (2004). Urocortin 1 in colonic mucosa in patients with ulcerative colitis. J. Clin. Endocrinol. Metab..

[107] Fukudo S. (2007). Role of corticotropin-releasing hormone in irritable bowel syndrome and intestinal inflammation. J. Gastroenterol..

[108] Im E., Rhee S.H., Park Y.S., Fiocchi C., Taché Y., Pothoulakis C. (2010). Corticotropin-releasing hormone family of peptides regulates intestinal angiogenesis. Gastroenterology.

[109] Kokkotou E., Torres D., Moss A.C., O’Brien M., Grigoriadis D.E., Karalis K., Pothoulakis C. (2006). Corticotropin-releasing hormone receptor 2-deficient mice have reduced intestinal inflammatory responses. J. Immunol..

[110] Reubi J.C., Waser B., Vale W., Rivier J. (2003). Expression of CRF1 and CRF2 receptors in human cancers. J. Clin. Endocrinol. Metab..

[111] Dermitzaki E., Tsatsanis C., Minas V., Chatzaki E., Charalampopoulos I., Venihaki M., Androulidaki A., Lambropoulou M., Spiess J., Michalodimitrakis E. (2007). Corticotropin-releasing factor (CRF) and the urocortins differentially regulate catecholamine secretion in human and rat adrenals, in a CRF receptor type-specific manner. Endocrinology.

[112] Hughes P.A., Zola H., Penttila I.A., Blackshaw L.A., Andrews J.M., Krumbiegel D. (2013). Immune activation in irritable bowel syndrome: Can neuroimmune interactions explain symptoms?. Am. J. Gastroenterol..

[113] Hu Y., Li M., Lu B., Wang X., Chen C., Zhang M. (2016). Corticotropin-releasing factor augments LPS-induced immune/inflammatory responses in JAWSII cells. Immunol. Res..

[114] Stengel A., Taché Y. (2010). Corticotropin-releasing factor signaling and visceral response to stress. Exp. Biol. Med..

[115] Shih D.Q., Targan S.R. (2008). Immunopathogenesis of inflammatory bowel disease. World J. Gastroenterol..

[116] Dunlop S.P., Jenkins D., Spiller R.C. (2003). Distinctive clinical, psychological, and histological features of postinfective irritable bowel syndrome. Am. J. Gastroenterol..

[117] Spiller R.C., Jenkins D., Thornley J.P., Hebden J.M., Wright T., Skinner M., Neal K.R. (2000). Increased rectal mucosal enteroendocrine cells, T lymphocytes, and increased gut permeability following acute Campylobacter enteritis and in post-dysenteric irritable bowel syndrome. Gut.

[118] Vermillion D.L., Ernst P.B., Collins S.M. (1991). T-lymphocyte modulation of intestinal muscle function in the Trichinella-infected rat. Gastroenterology.

[119] Ohman L., Lindmark A.-C., Isaksson S., Posserud I., Strid H., Sjövall H., Simrén M. (2009). B-cell activation in patients with irritable bowel syndrome (IBS). Neurogastroenterol. Motil..

[120] Forshammar J., Isaksson S., Strid H., Stotzer P.-O., Sjövall H., Simrén M., Ohman L. (2008). A pilot study of colonic B cell pattern in irritable bowel syndrome. Scand. J. Gastroenterol..

[121] Baker C., Richards L.J., Dayan C.M., Jessop D.S. (2003). Corticotropin-releasing hormone immunoreactivity in human T and B cells and macrophages: Colocalization with arginine vasopressin. J. Neuroendocrinol..

[122] Yuan P.-Q., Wu S.V., Elliott J., Anton P.A., Chatzaki E., Million M., Taché Y. (2012). Expression of corticotropin releasing factor receptor type 1 (CRF1) in the human gastrointestinal tract and upregulation in the colonic mucosa in patients with ulcerative colitis. Peptides.

[123] Arseneau K.O., Tamagawa H., Pizarro T.T., Cominelli F. (2007). Innate and adaptive immune responses related to IBD pathogenesis. Curr. Gastroenterol. Rep..

[124] Frdrichie M., Pohin M., Powrie F. (2019). Cytokine Networks in the Pathophysiology of Inflammatory Bowel Disease. Immunity.

[125] Singh V.K., Leu S.J. (1990). Enhancing effect of corticotropin-releasing neurohormone on the production of interleukin-1 and interleukin-2. Neurosci. Lett..

[126] Ekman R., Servenius B., Castro M.G., Lowry P.J., Cederlund A.S., Bergman O., Sjögren H.O. (1993). Biosynthesis of corticotropin-releasing hormone in human T-lymphocytes. J. Neuroimmunol..

[127] Kravchenco I.V., Furalev V.A. (1994). Secretion of immunoreactive corticotropin releasing factor and adrenocorticotropic hormone by T- and B-lymphocytes in response to cellular stress factors. Biochem. Biophys. Res. Commun..

[128] Gravanis A., Margioris A.N. (2005). The corticotropin-releasing factor (CRF) family of neuropeptides in inflammation: Potential therapeutic applications. Curr. Med. Chem..

[129] Huang M., Kempuraj D., Papadopoulou N., Kourelis T., Donelan J., Manola A., Theoharides T.C. (2009). Urocortin induces interleukin-6 release from rat cardiomyocytes through p38 MAP kinase, ERK and NF-kappaB activation. J. Mol. Endocrinol..

[130] Gay J., Kokkotou E., O’Brien M., Pothoulakis C., Karalis K.P. (2008). Corticotropin-releasing hormone deficiency is associated with reduced local inflammation in a mouse model of experimental colitis. Endocrinology.

[131] Anton P.M., Gay J., Mykoniatis A., Pan A., O’Brien M., Brown D., Karalis K., Pothoulakis C. (2004). Corticotropin-releasing hormone (CRH) requirement in Clostridium difficile toxin A-mediated intestinal inflammation. Proc. Natl. Acad. Sci. USA.

[132] Gonzalez-Rey E., Chorny A., Varela N., Robledo G., Delgado M. (2006). Urocortin and adrenomedullin prevent lethal endotoxemia by down-regulating the inflammatory response. Am. J. Pathol..

[133] Gonzalez-Rey E., Fernandez-Martin A., Chorny A., Delgado M. (2006). Therapeutic effect of urocortin and adrenomedullin in a murine model of Crohn’s disease. Gut.

[134] Meng L., Lu Z., Xiaoteng W., Yue H., Bin L., Lina M., Zhe C. (2015). Corticotropin-releasing Factor Changes the Phenotype and Function of Dendritic Cells in Mouse Mesenteric Lymph Nodes. J. Neurogastroenterol. Motil..

[135] Hojo M., Ohkusa T., Tomeoku H., Koido S., Asaoka D., Nagahara A., Watanabe S. (2011). Corticotropin-releasing factor secretion from dendritic cells stimulated by commensal bacteria. World J. Gastroenterol..

[136] Koido S., Ohkusa T., Kan S., Takakura K., Saito K., Komita H., Ito Z., Kobayashi H., Takami S., Uchiyama K. (2014). Production of corticotropin-releasing factor and urocortin from human monocyte-derived dendritic cells is stimulated by commensal bacteria in intestine. World J. Gastroenterol..

[137] Lee H.J., Kwon Y.S., Park C.O., Oh S.H., Lee J.H., Wu W.H., Chang N.S., Lee M.-G., Lee K.H. (2009). Corticotropin-releasing factor decreases IL-18 in the monocyte-derived dendritic cell. Exp. Dermatol..

[138] Zhuang Z., Zhang L., Wang X., Tao L., Lv B. (2016). PDIA3 gene induces visceral hypersensitivity in rats with irritable bowel syndrome through the dendritic cell-mediated activation of T cells. PeerJ.

[139] Long Y., Wang W., Wang H., Hao L., Qian W., Hou X. (2012). Characteristics of intestinal lamina propria dendritic cells in a mouse model of postinfectious irritable bowel syndrome. J. Gastroenterol. Hepatol..

[140] Tsatsanis C., Androulidaki A., Alissafi T., Charalampopoulos I., Dermitzaki E., Roger T., Gravanis A., Margioris A.N. (2006). Corticotropin-releasing factor and the urocortins induce the expression of TLR4 in macrophages via activation of the transcription factors PU.1 and AP-1. J. Immunol..

[141] Smith E.M., Gregg M., Hashemi F., Schott L., Hughes T.K. (2006). Corticotropin Releasing Factor (CRF) activation of NF-kappaB-directed transcription in leukocytes. Cell. Mol. Neurobiol..

[142] Vanner S., Greenwood-Van Meerveld B., Mawe G., Shea-Donohue T., Verdu E.F., Wood J., Grundy D. (2016). Fundamentals of Neurogastroenterology: Basic Science. Gastroenterology.

[143] Muller P.A., Koscsó B., Rajani G.M., Stevanovic K., Berres M.-L., Hashimoto D., Mortha A., Leboeuf M., Li X.-M., Mucida D. (2014). Crosstalk between muscularis macrophages and enteric neurons regulates gastrointestinal motility. Cell.

[144] Agelaki S., Tsatsanis C., Gravanis A., Margioris A.N. (2002). Corticotropin-releasing hormone augments proinflammatory cytokine production from macrophages *in vitro* and in lipopolysaccharide-induced endotoxin shock in mice. Infect. Immun..

[145] Black P.H. (2002). Stress and the inflammatory response: A review of neurogenic inflammation. Brain Behav. Immun..

[146] Tsatsanis C., Androulidaki A., Dermitzaki E., Gravanis A., Margioris A.N. (2007). Corticotropin releasing factor receptor 1 (CRF1) and CRF2 agonists exert an anti-inflammatory effect during the early phase of inflammation suppressing LPS-induced TNF-alpha release from macrophages via induction of COX-2 and PGE2. J. Cell. Physiol..

[147] Koshida H., Kotake Y. (1994). Corticotropin-releasing hormone enhances the superoxide anion production of rabbit peritoneal macrophages stimulated with N-formyl-methionyl-leucyl-phenylalanine. Life Sci..

[148] Tsatsanis C., Androulidaki A., Dermitzaki E., Charalampopoulos I., Spiess J., Gravanis A., Margioris A.N. (2005). Urocortin 1 and Urocortin 2 induce macrophage apoptosis via CRFR2. FEBS Lett..

[149] Chaniotou Z., Giannogonas P., Theoharis S., Teli T., Gay J., Savidge T., Koutmani Y., Brugni J., Kokkotou E., Pothoulakis C. (2010). Corticotropin-releasing factor regulates TLR4 expression in the colon and protects mice from colitis. Gastroenterology.

[150] Patsos G., Corfield A. (2009). Management of the human mucosal defensive barrier: Evidence for glycan legislation. Biol. Chem..

[151] Huttner K.M., Bevins C.L. (1999). Antimicrobial peptides as mediators of epithelial host defense. Pediatr. Res..

[152] Kawahito Y., Sano H., Mukai S., Asai K., Kimura S., Yamamura Y., Kato H., Chrousos G.P., Wilder R.L., Kondo M. (1995). Corticotropin releasing hormone in colonic mucosa in patients with ulcerative colitis. Gut.

[153] Chatzaki E., Crowe P.D., Wang L., Million M., Taché Y., Grigoriadis D.E. (2004). CRF receptor type 1 and 2 expression and anatomical distribution in the rat colon. J. Neurochem..

[154] la Fleur S.E., Wick E.C., Idumalla P.S., Grady E.F., Bhargava A. (2005). Role of peripheral corticotropin-releasing factor and urocortin II in intestinal inflammation and motility in terminal ileum. Proc. Natl. Acad. Sci. USA.

[155] Castagliuolo I., Lamont J.T., Qiu B., Fleming S.M., Bhaskar K.R., Nikulasson S.T., Kornetsky C., Pothoulakis C. (1996). Acute stress causes mucin release from rat colon: Role of corticotropin releasing factor and mast cells. Am. J. Physiol..

[156] Pfeiffer C.J., Qiu B., Lam S.K. (2001). Reduction of colonic mucus by repeated short-term stress enhances experimental colitis in rats. J. Physiol. Paris.

[157] Söderholm J.D., Yang P.-C., Ceponis P., Vohra A., Riddell R., Sherman P.M., Perdue M.H. (2002). Chronic stress induces mast cell-dependent bacterial adherence and initiates mucosal inflammation in rat intestine. Gastroenterology.

[158] Lee S.H. (2015). Intestinal permeability regulation by tight junction: Implication on inflammatory bowel diseases. Intest. Res..

[159] Yue H., Bin L., Chaoying C., Meng Z., Meng L., Xi W. (2017). Potential Regulatory Effects of Corticotropin-Releasing Factor on Tight Junction-Related Intestinal Epithelial Permeability are Partially Mediated by CK8 Upregulation. Cell. Physiol. Biochem..

[160] Zhang L., Song J., Bai T., Qian W., Hou X.-H. (2017). Stress induces more serious barrier dysfunction in follicle-associated epithelium than villus epithelium involving mast cells and protease-activated receptor-2. Sci. Rep..

[161] Yu Y., Liu Z.-Q., Liu X.-Y., Yang L., Geng X.-R., Yang G., Liu Z.-G., Zheng P.-Y., Yang P.-C. (2013). Stress-Derived Corticotropin Releasing Factor Breaches Epithelial Endotoxin Tolerance. PLoS ONE.

[162] Jizhong S., Qiaomin W., Chao W., Yanqing L. (2016). Corticotropin-Releasing Factor and Toll-Like Receptor Gene Expression Is Associated with Low-Grade Inflammation in Irritable Bowel Syndrome Patients with Depression. Gastroenterol. Res. Pract..

[163] Santos J., Yates D., Guilarte M., Vicario M., Alonso C., Perdue M.H. (2008). Stress neuropeptides evoke epithelial responses via mast cell activation in the rat colon. Psychoneuroendocrinology.

[164] Hoffman J.M., Baritaki S., Ruiz J.J., Sideri A., Pothoulakis C. (2016). Corticotropin-Releasing Hormone Receptor 2 Signaling Promotes Mucosal Repair Responses after Colitis. Am. J. Pathol..

[165] Schoultz I., Keita Å. (2019). Cellular and Molecular Therapeutic Targets in Inflammatory Bowel Disease—Focusing on Intestinal Barrier Function. Cells.

[166] Barreau F., Cartier C., Leveque M., Ferrier L., Moriez R., Laroute V., Rosztoczy A., Fioramonti J., Bueno L. (2007). Pathways involved in gut mucosal barrier dysfunction induced in adult rats by maternal deprivation: Corticotrophin-releasing factor and nerve growth factor interplay. J. Physiol..

[167] Cao J., Papadopoulou N., Kempuraj D., Boucher W.S., Sugimoto K., Cetrulo C.L., Theoharides T.C. (2005). Human mast cells express corticotropin-releasing hormone (CRH) receptors and CRH leads to selective secretion of vascular endothelial growth factor. J. Immunol..

[168] Ohman L., Isaksson S., Lundgren A., Simrén M., Sjövall H. (2005). A controlled study of colonic immune activity and beta7+ blood T lymphocytes in patients with irritable bowel syndrome. Clin. Gastroenterol. Hepatol..

[169] Gebhardt T., Lorentz A., Detmer F., Trautwein C., Bektas H., Manns M.P., Bischoff S.C. (2005). Growth, phenotype, and function of human intestinal mast cells are tightly regulated by transforming growth factor beta1. Gut.

[170] Wallon C., Yang P.-C., Keita A.V., Ericson A.-C., McKay D.M., Sherman P.M., Perdue M.H., Söderholm J.D. (2008). Corticotropin-releasing hormone (CRH) regulates macromolecular permeability via mast cells in normal human colonic biopsies *in vitro*. Gut.

[171] Saunders P.R., Santos J., Hanssen N.P.M., Yates D., Groot J.A., Perdue M.H. (2002). Physical and psychological stress in rats enhances colonic epithelial permeability via peripheral CRH. Dig. Dis. Sci..

[172] Overman E.L., Rivier J.E., Moeser A.J. (2012). CRF induces intestinal epithelial barrier injury via the release of mast cell proteases and TNF-α. PLoS ONE.

[173] Wilcz-Villega E.M., McClean S., O’Sullivan M.A. (2013). Mast cell tryptase reduces junctional adhesion molecule-A (JAM-A) expression in intestinal epithelial cells: Implications for the mechanisms of barrier dysfunction in irritable bowel syndrome. Am. J. Gastroenterol..

[174] Zheng P.-Y., Feng B.-S., Oluwole C., Struiksma S., Chen X., Li P., Tang S.-G., Yang P.-C. (2009). Psychological stress induces eosinophils to produce corticotrophin releasing hormone in the intestine. Gut.

[175] Vanuytsel T., van Wanrooy S., Vanheel H., Vanormelingen C., Verschueren S., Houben E., Salim Rasoel S., Tόth J., Holvoet L., Farré R. (2014). Psychological stress and corticotropin-releasing hormone increase intestinal permeability in humans by a mast cell-dependent mechanism. Gut.

[176] Cameron H.L., Perdue M.H. (2005). Stress impairs murine intestinal barrier function: Improvement by glucagon-like peptide-2. J. Pharmacol. Exp. Ther..

[177] Söderholm J.D., Perdue M.H. (2001). Stress and gastrointestinal tract. II. Stress and intestinal barrier function. Am. J. Physiol. Gastrointest. Liver Physiol..

[178] Bale T.L., Giordano F.J., Hickey R.P., Huang Y., Nath A.K., Peterson K.L., Vale W.W., Lee K.-F. (2002). Corticotropin-releasing factor receptor 2 is a tonic suppressor of vascularization. Proc. Natl. Acad. Sci. USA.

[179] Karalis K., Sano H., Redwine J., Listwak S., Wilder R.L., Chrousos G.P. (1991). Autocrine or paracrine inflammatory actions of corticotropin-releasing hormone in vivo. Science.

[180] Simoncini T., Apa R., Reis F.M., Miceli F., Stomati M., Driul L., Lanzone A., Genazzani A.R., Petraglia F. (1999). Human umbilical vein endothelial cells: A new source and potential target for corticotropin-releasing factor. J. Clin. Endocrinol. Metab..

[181] Cantarella G., Lempereur L., Lombardo G., Chiarenza A., Pafumi C., Zappalà G., Bernardini R. (2001). Divergent effects of corticotropin releasing hormone on endothelial cell nitric oxide synthase are associated with different expression of CRH type 1 and 2 receptors. Br. J. Pharmacol..

[182] Porcher C., Juhem A., Peinnequin A., Sinniger V., Bonaz B. (2005). Expression and effects of metabotropic CRF1 and CRF2 receptors in rat small intestine. Am. J. Physiol. Gastrointest. Liver Physiol..

[183] Gao X., Cao Q., Cheng Y., Zhao D., Wang Z., Yang H., Wu Q., You L., Wang Y., Lin Y. (2018). Chronic stress promotes colitis by disturbing the gut microbiota and triggering immune system response. Proc. Natl. Acad. Sci. USA.

[184] Sun Y., Zhang M., Chen C.-C., Gillilland M., Sun X., El-Zaatari M., Huffnagle G.B., Young V.B., Zhang J., Hong S.-C. (2013). Stress-induced corticotropin-releasing hormone-mediated NLRP6 inflammasome inhibition and transmissible enteritis in mice. Gastroenterology.

[185] Chang Y.-M., El-Zaatari M., Kao J.Y. (2014). Does stress induce bowel dysfunction?. Expert Rev. Gastroenterol. Hepatol..

[186] Xu D., Gao J., Gillilland M., Wu X., Song I., Kao J.Y., Owyang C. (2014). Rifaximin alters intestinal bacteria and prevents stress-induced gut inflammation and visceral hyperalgesia in rats. Gastroenterology.

[187] Ait-Belgnaoui A., Han W., Lamine F., Eutamene H., Fioramonti J., Bueno L., Theodorou V. (2006). Lactobacillus farciminis treatment suppresses stress induced visceral hypersensitivity: A possible action through interaction with epithelial cell cytoskeleton contraction. Gut.

[188] Eutamene H., Lamine F., Chabo C., Theodorou V., Rochat F., Bergonzelli G.E., Corthésy-Theulaz I., Fioramonti J., Bueno L. (2007). Synergy between Lactobacillus paracasei and its bacterial products to counteract stress-induced gut permeability and sensitivity increase in rats. J. Nutr..

[189] Carding S., Verbeke K., Vipond D.T., Corfe B.M., Owen L.J. (2015). Dysbiosis of the gut microbiota in disease. Microb. Ecol. Health Dis..

[190] Noor S.O., Ridgway K., Scovell L., Kemsley E.K., Lund E.K., Jamieson C., Johnson I.T., Narbad A. (2010). Ulcerative colitis and irritable bowel patients exhibit distinct abnormalities of the gut microbiota. BMC Gastroenterol..

[191] Moayyedi P., Ford A.C., Talley N.J., Cremonini F., Foxx-Orenstein A.E., Brandt L.J., Quigley E.M.M. (2010). The efficacy of probiotics in the treatment of irritable bowel syndrome: A systematic review. Gut.

[192] Dai C., Zheng C.-Q., Jiang M., Ma X.-Y., Jiang L.-J. (2013). Probiotics and irritable bowel syndrome. World J. Gastroenterol..

[193] Murakami T., Kamada K., Mizushima K., Higashimura Y., Katada K., Uchiyama K., Handa O., Takagi T., Naito Y., Itoh Y. (2017). Changes in Intestinal Motility and Gut Microbiota Composition in a Rat Stress Model. Digestion.

[194] Martin C.R., Osadchiy V., Kalani A., Mayer E.A. (2018). The Brain-Gut-Microbiome Axis. Cell. Mol. Gastroenterol. Hepatol..

[195] Holzer P., Farzi A. (2014). Neuropeptides and the microbiota–gut–brain axis. Adv. Exp. Med. Biol..

[196] Ducarouge B., Pelissier-Rota M., Lainé M., Cristina N., Vachez Y., Scoazec J.-Y., Bonaz B., Jacquier-Sarlin M. (2013). CRF2 signaling is a novel regulator of cellular adhesion and migration in colorectal cancer cells. PLoS ONE.

[197] Rodriguez J.A., Huerta-Yepez S., Law I.K.M., Baay-Guzman G.J., Tirado-Rodriguez B., Hoffman J.M., Iliopoulos D., Hommes D.W., Verspaget H.W., Chang L. (2015). Diminished expression of CRHR2 in human colon cancer promotes tumor growth and EMT via persistent IL-6/Stat3 signaling. Cell. Mol. Gastroenterol. Hepatol..

[198] Mueller M.M., Fusenig N.E. (2004). Friends or foes—Bipolar effects of the tumour stroma in cancer. Nat. Rev. Cancer.

[199] Kageyama K., Hanada K., Nigawara T., Moriyama T., Terui K., Sakihara S., Suda T. (2006). Urocortin induces interleukin-6 gene expression via cyclooxygenase-2 activity in aortic smooth muscle cells. Endocrinology.

[200] Jarnicki A., Putoczki T., Ernst M. (2010). Stat3: Linking inflammation to epithelial cancer—More than a “gut” feeling?. Cell Div..

[201] Klampfer L. (2008). The role of signal transducers and activators of transcription in colon cancer. Front. Biosci..

[202] Waldner M.J., Foersch S., Neurath M.F. (2012). Interleukin-6--a key regulator of colorectal cancer development. Int. J. Biol. Sci..

[203] Fang X., Hong Y., Dai L., Qian Y., Zhu C., Wu B., Li S. (2017). CRH promotes human colon cancer cell proliferation via IL-6/JAK2/STAT3 signaling pathway and VEGF-induced tumor angiogenesis. Mol. Carcinog..

[204] Liu Y., Fang X., Yuan J., Sun Z., Li C., Li R., Li L., Zhu C., Wan R., Guo R. (2014). The role of corticotropin-releasing hormone receptor 1 in the development of colitis-associated cancer in mouse model. Endocr. Relat. Cancer.

[205] Pelissier-Rota M., Chartier N.T., Bonaz B., Jacquier-Sarlin M.R. (2017). A crosstalk between muscarinic and CRF2 receptors regulates cellular adhesion properties of human colon cancer cells. Biochim. Biophys. Acta Mol. Cell Res..

[206] Ducarouge B., Pelissier-Rota M., Powell R., Buisson A., Bonaz B., Jacquier-Sarlin M. (2017). Involvement of CRF2 signaling in enterocyte differentiation. World J. Gastroenterol..

[207] Pothoulakis C., Torre-Rojas M., Duran-Padilla M.A., Gevorkian J., Zoras O., Chrysos E., Chalkiadakis G., Baritaki S. (2018). CRHR2/Ucn2 signaling is a novel regulator of miR-7/YY1/Fas circuitry contributing to reversal of colorectal cancer cell resistance to Fas-mediated apoptosis. Int. J. Cancer.

[208] Zhang N., Li X., Wu C.W., Dong Y., Cai M., Mok M.T.S., Wang H., Chen J., Ng S.S.M., Chen M. (2013). microRNA-7 is a novel inhibitor of YY1 contributing to colorectal tumorigenesis. Oncogene.

